# Supplementary material for: Click beetle luciferase mutant and near infrared naphthyl-luciferins for improved bioluminescence imaging
Source: Nat Commun. 2018 Jan 9;9:132. doi: 10.1038/s41467-017-02542-9 (PMC5760652; doi:10.1038/s41467-017-02542-9)
Supplement: Supplementary file 1 — Supplementary Information [file 41467_2017_2542_MOESM1_ESM.pdf]

(a)

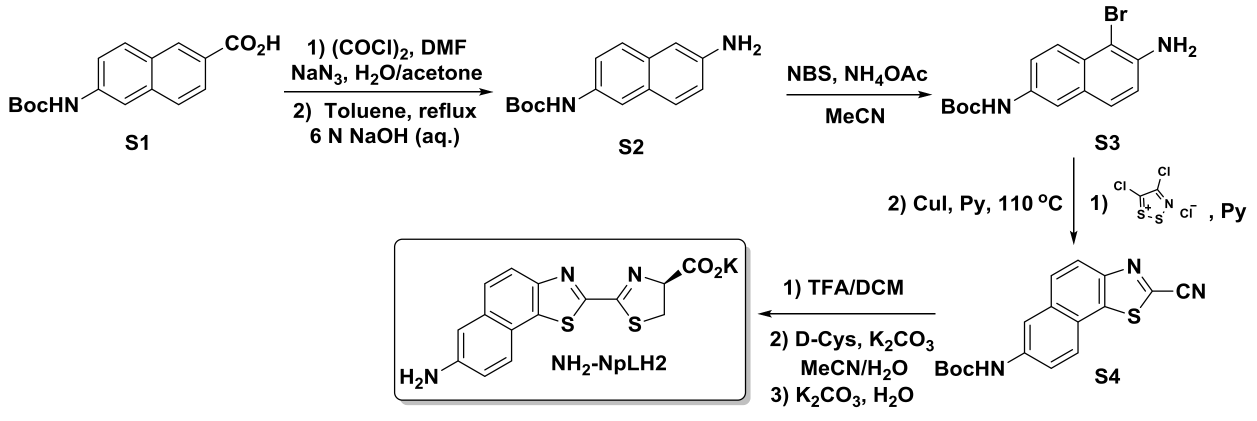

(b)

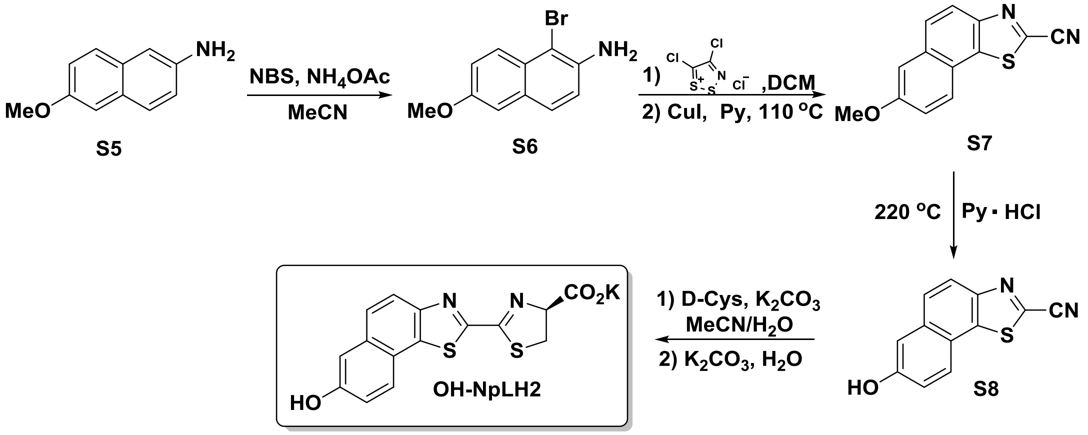

Supplementary Figure 1. Synthesis of NIR naphthyl-luciferin substrates. (**a**) Scheme of NH_2-NpLH2 synthesis. (**b**) Scheme of OH-NpLH2 synthesis.

(a)

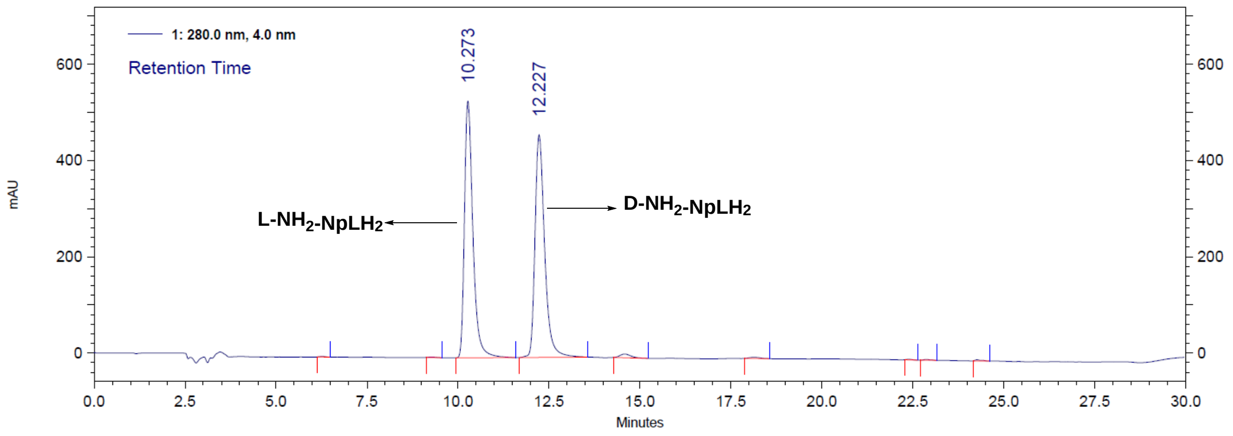

1: 280.0 nm, 4.0 nm
Retention Time
10.273
12.227
L-NH$_2$-NpLH$_2$
D-NH$_2$-NpLH$_2$
mAU
Minutes

(b)

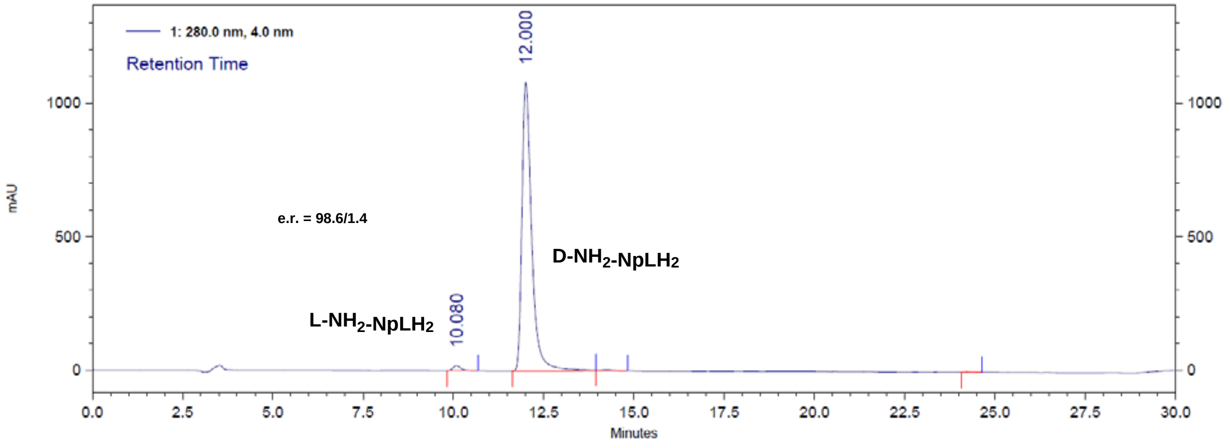

1: 280.0 nm, 4.0 nm
Retention Time
12.000
e.r. = 98.6/1.4
D-NH$_2$-NpLH$_2$
L-NH$_2$-NpLH$_2$
10.080
mAU
Minutes

(c)

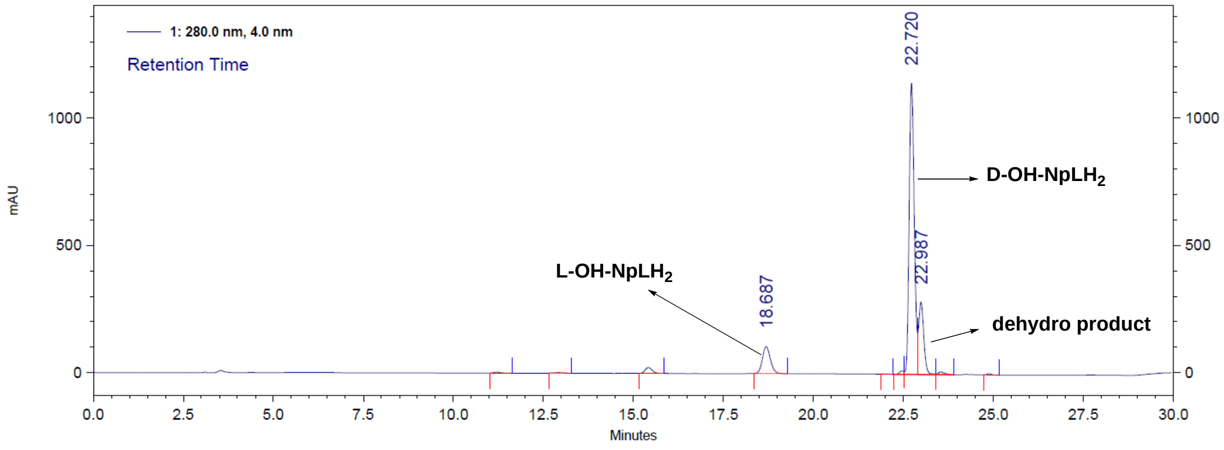

1: 280.0 nm, 4.0 nm
Retention Time
22.720
D-OH-NpLH$_2$
22.987
L-OH-NpLH$_2$
18.687
dehydro product
mAU
Minutes

(d)

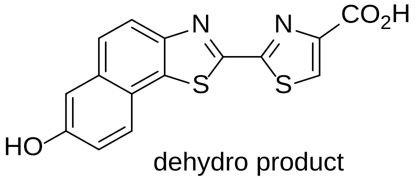

(e)

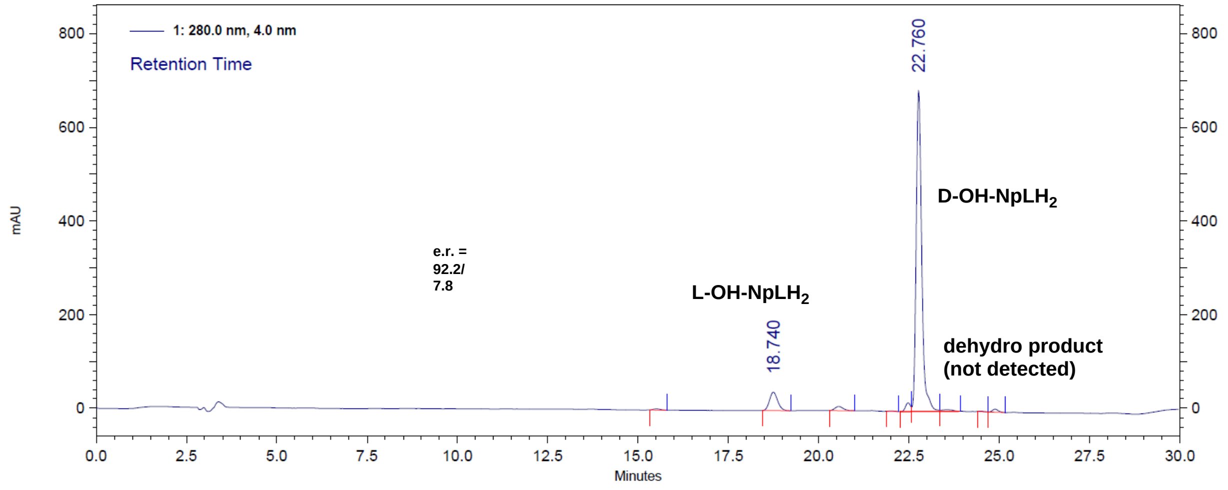

Supplementary Figure 2. Enantiopurity analysis. (**a**) Chiral HPLC trace of racemic NH_2-NpLH2. (**b**) Chiral HPLC trace of NH_2-NpLH2 demonstrating high enantiopurity. (**c**) Chiral HPLC trace of matured stock of OH-NpLH2 in H_2O. 100 µM of D-OH-NpLH2 with high enantiopurity in H_2O was incubated at 60 °C for 24 h to induce racemization. This also induced formation of the corresponding dehydro product (T_R=23 min). (**d**) Structure of dehydro product. (**e**) Chiral HPLC analysis of OH-NpLH2 showing high enantiopurity.

(a)

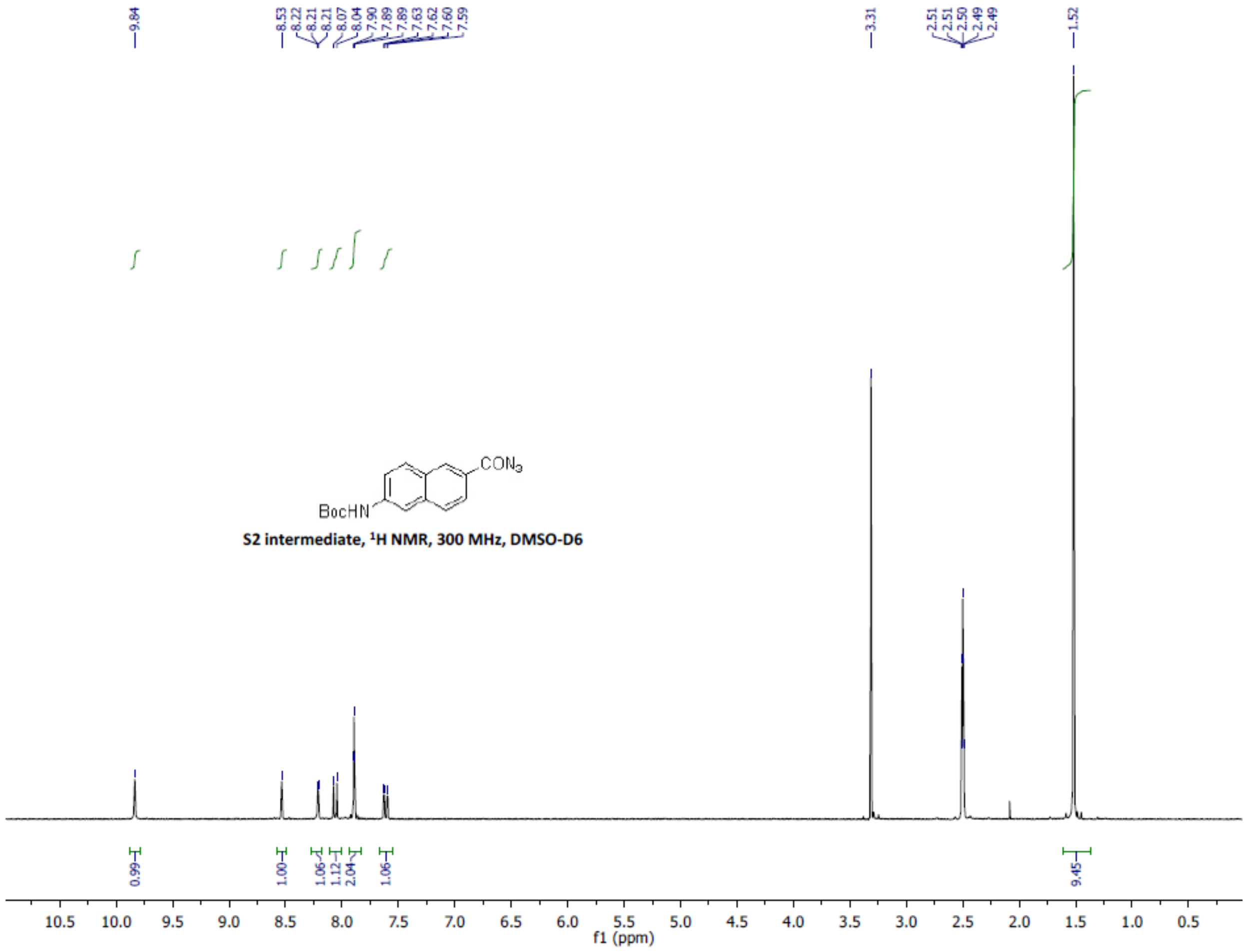

9.84
8.53 8.22 8.21 8.21 8.07 8.04 7.90 7.89 7.89 7.63 7.62 7.60 7.59
3.31
2.51 2.51 2.50 2.49 2.49
1.52
CON3
BocHN
S2 intermediate, 1H NMR, 300 MHz, DMSO-D6
0.99
1.00
1.06
1.12
2.04
1.06
9.45
10.5 10.0 9.5 9.0 8.5 8.0 7.5 7.0 6.5 6.0 5.5 5.0 4.5 4.0 3.5 3.0 2.5 2.0 1.5 1.0 0.5
f1 (ppm)

(b)

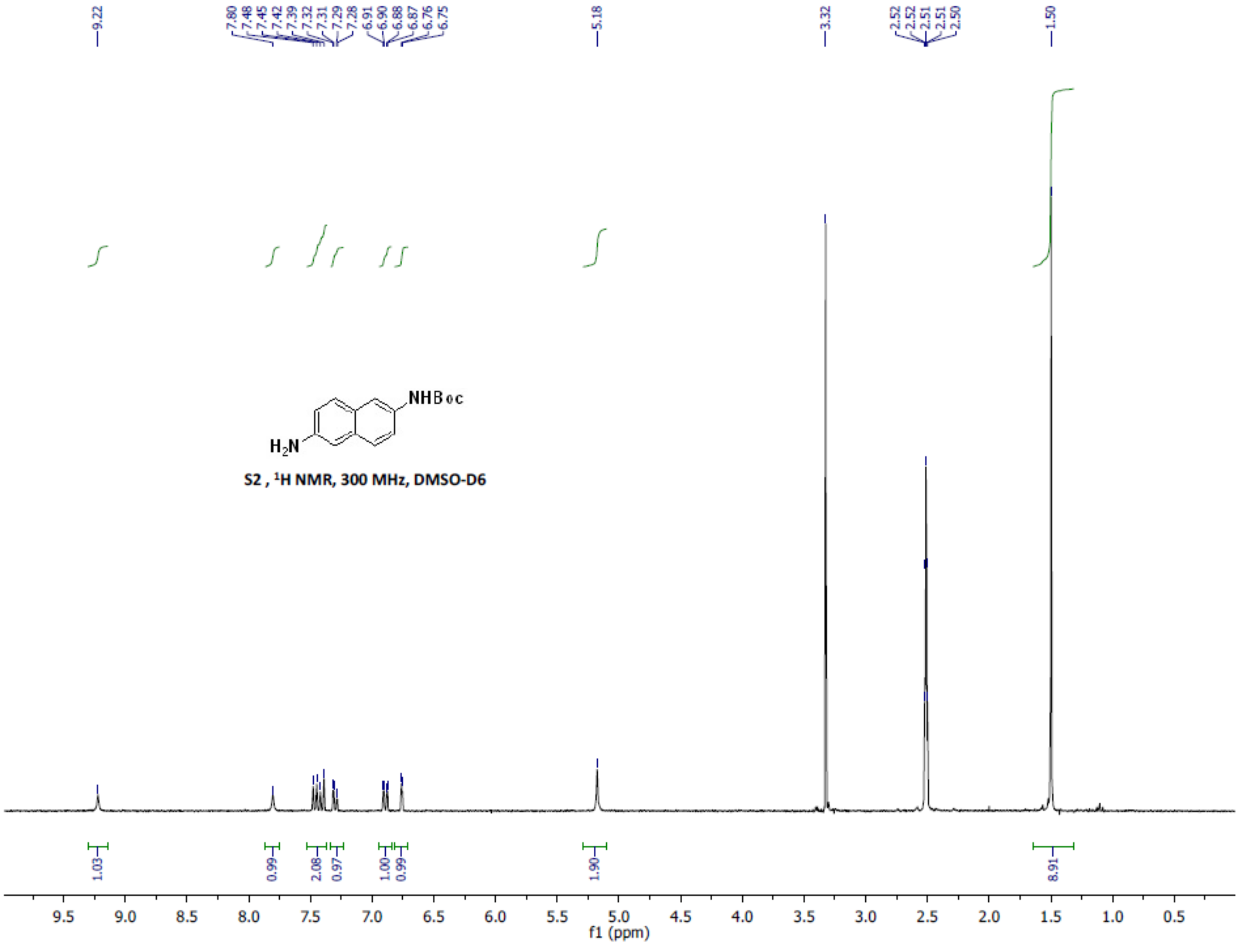
9.22
7.80
7.48
7.45
7.42
7.39
7.32
7.31
7.29
7.28
6.91
6.90
6.88
6.87
6.76
6.75
5.18
3.32
2.52
2.52
2.51
2.51
2.50
1.50
NHBoc
H2N
S2 , 1H NMR, 300 MHz, DMSO-D6
1.03
0.99
2.08
0.97
1.00
0.99
1.90
8.91
9.5
9.0
8.5
8.0
7.5
7.0
6.5
6.0
5.5
5.0
4.5
4.0
3.5
3.0
2.5
2.0
1.5
1.0
0.5
f1 (ppm)

(c)

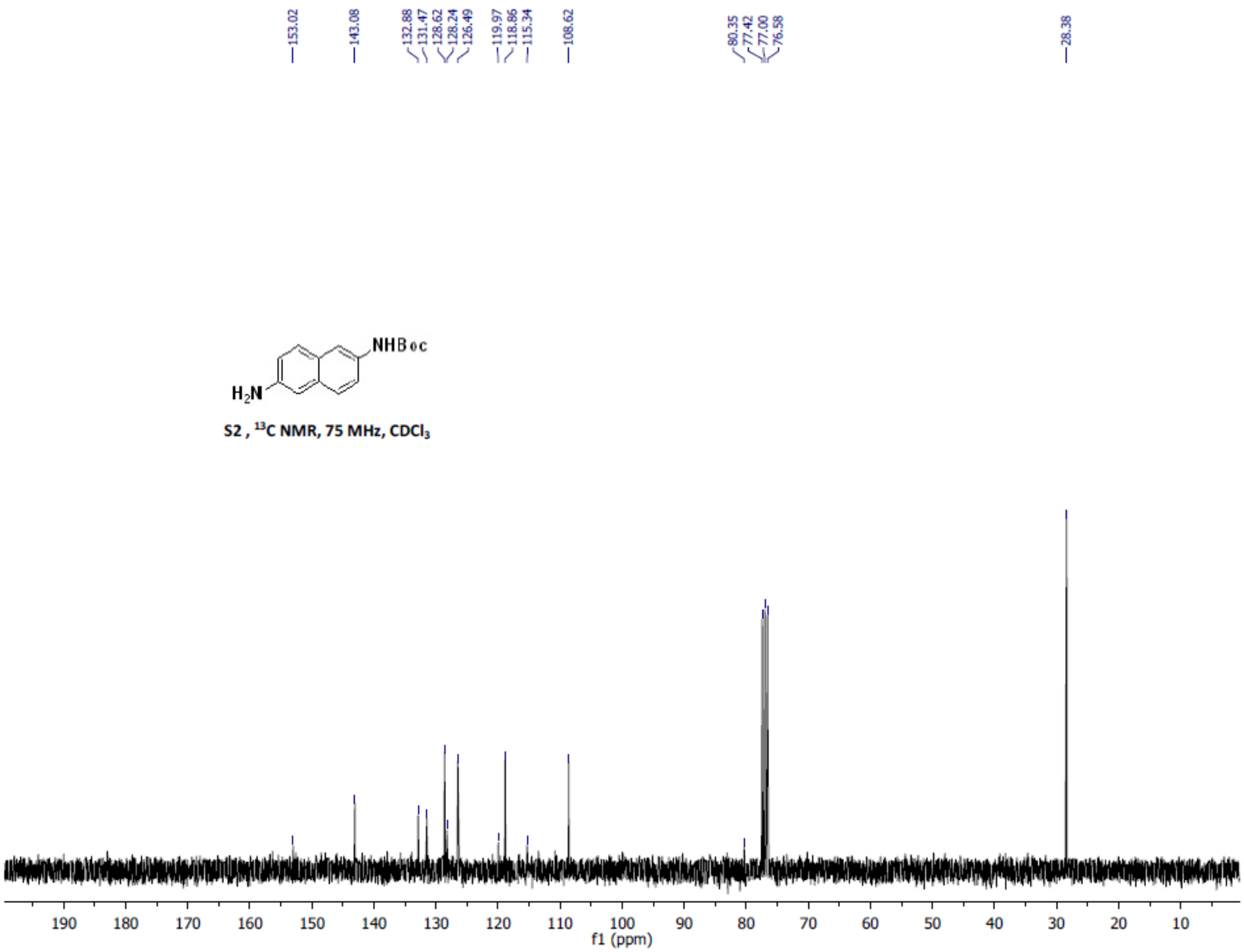

153.02
143.08
132.88
131.47
128.62
128.24
126.49
119.97
118.86
115.34
108.62
80.35
77.42
77.00
76.58
28.38
NHBoc
H2N
S2 , 13C NMR, 75 MHz, CDCl3
190
180
170
160
150
140
130
120
110
100
90
80
70
60
50
40
30
20
10
f1 (ppm)

(d)

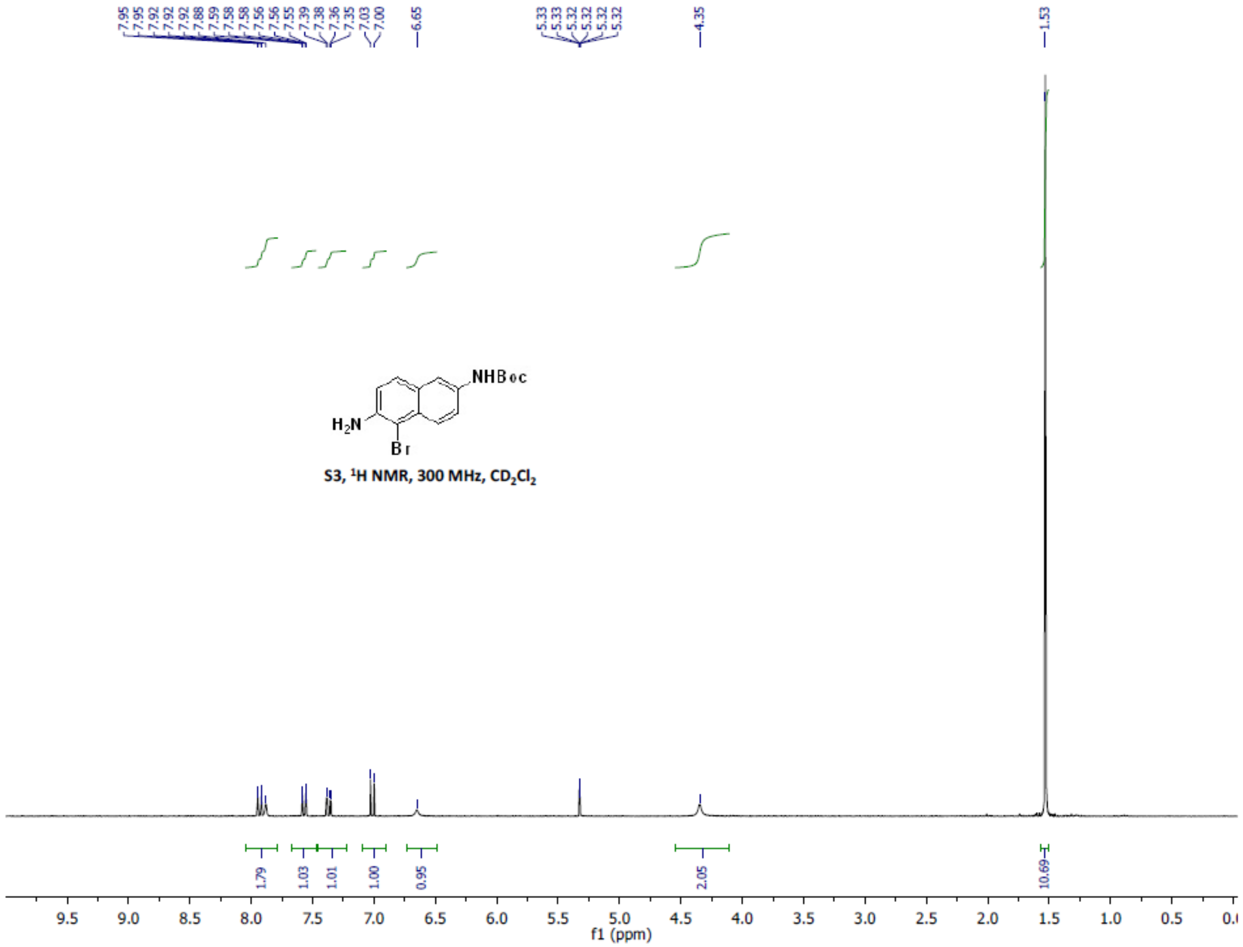

S3, ^{1}H NMR, 300 MHz, CD_2Cl_2

(e)

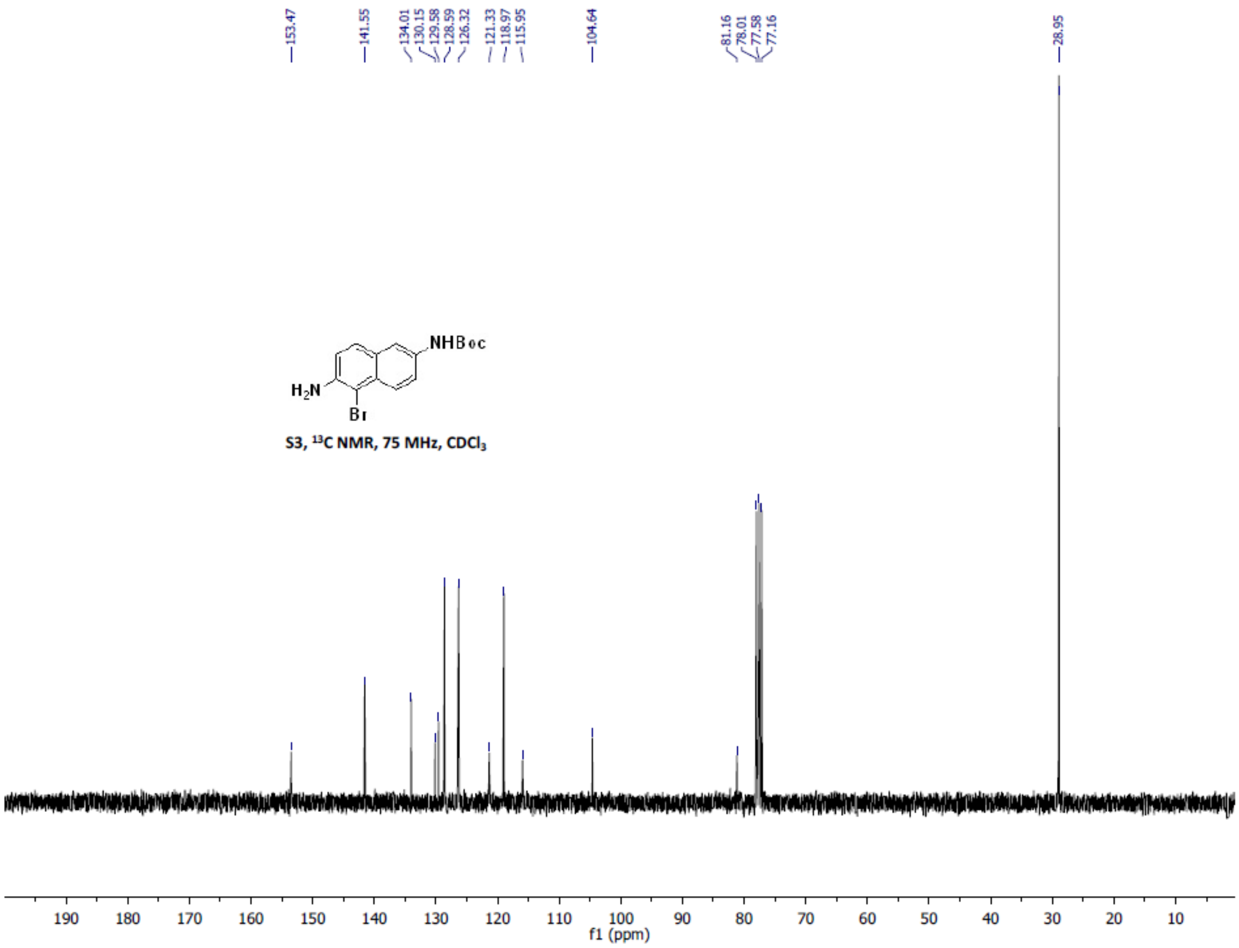
153.47
141.55
134.01
130.15
129.58
128.59
126.32
121.33
118.97
115.95
104.64
81.16
78.01
77.58
77.16
28.95
NHBoc
H2N
Br
S3, 13C NMR, 75 MHz, CDCl3
190 180 170 160 150 140 130 120 110 100 90 80 70 60 50 40 30 20 10
f1 (ppm)

(f)

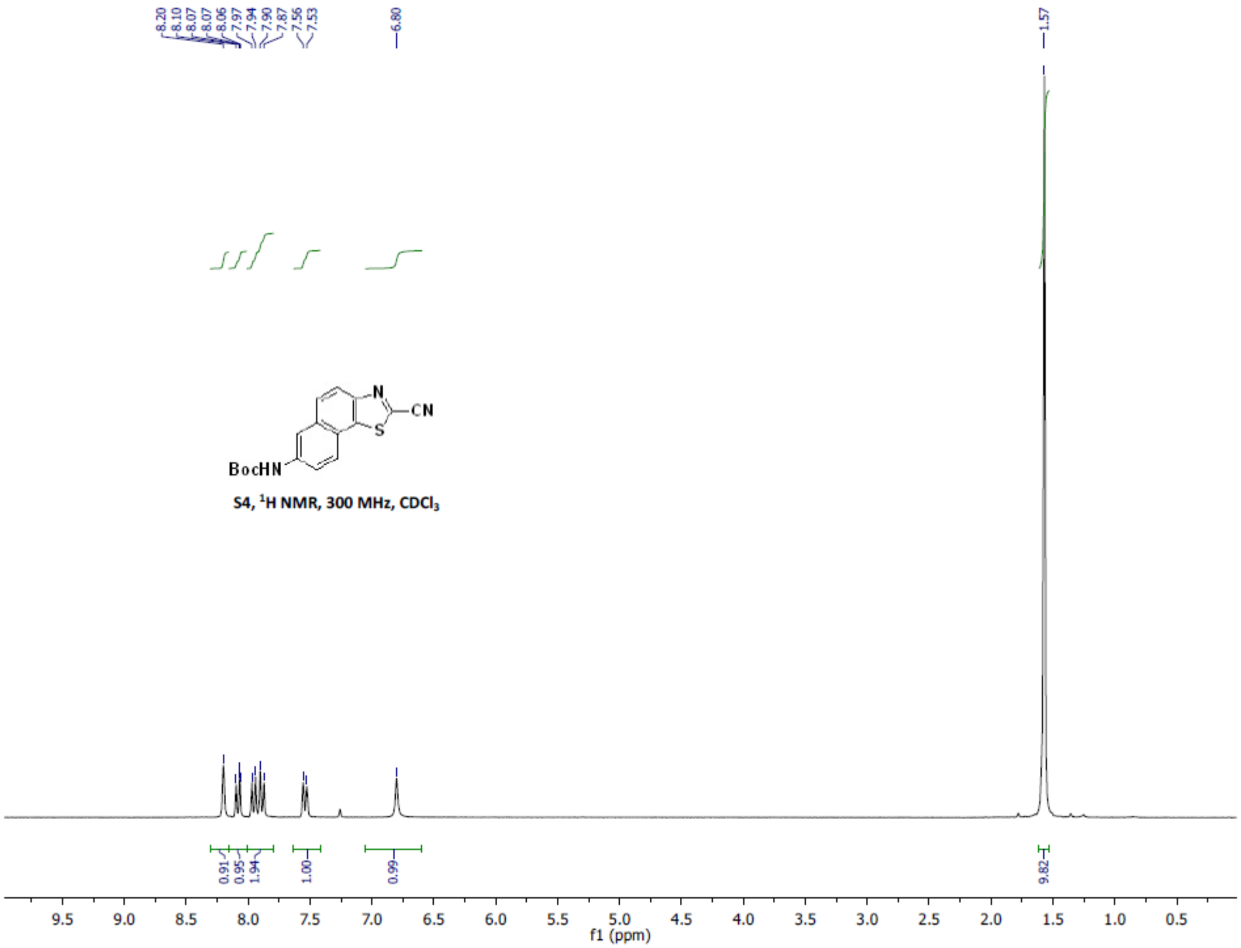

S4, ^{1}H NMR, 300 MHz, $CDCl_3$

(g)

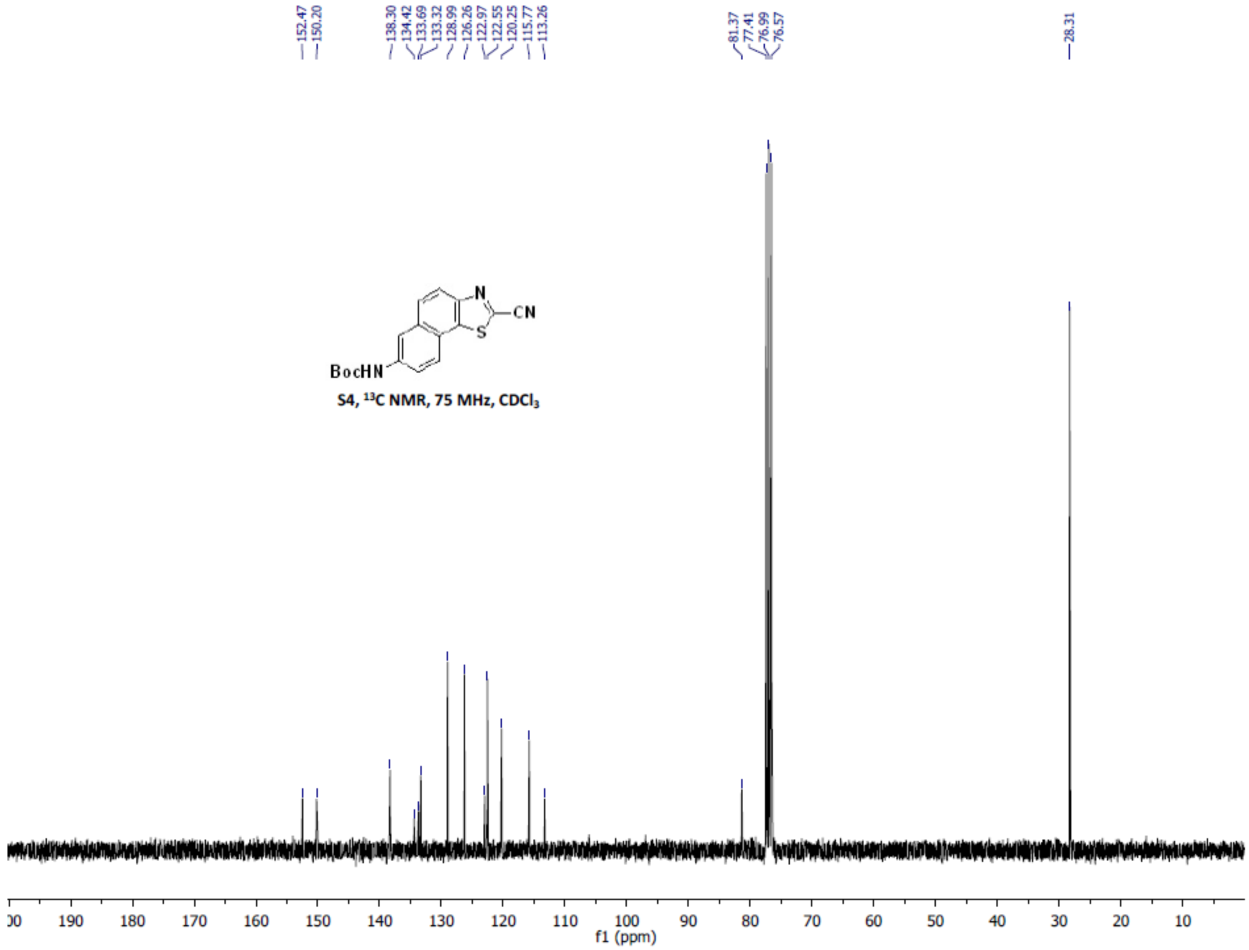
152.47
150.20
138.30
134.42
133.69
133.32
128.99
126.26
122.97
122.55
120.25
115.77
113.26
81.37
77.41
76.99
76.57
28.31
N
CN
S
BocHN
S4, ¹³C NMR, 75 MHz, CDCl₃
190 180 170 160 150 140 130 120 110 100 90 80 70 60 50 40 30 20 10
f1 (ppm)

(h)

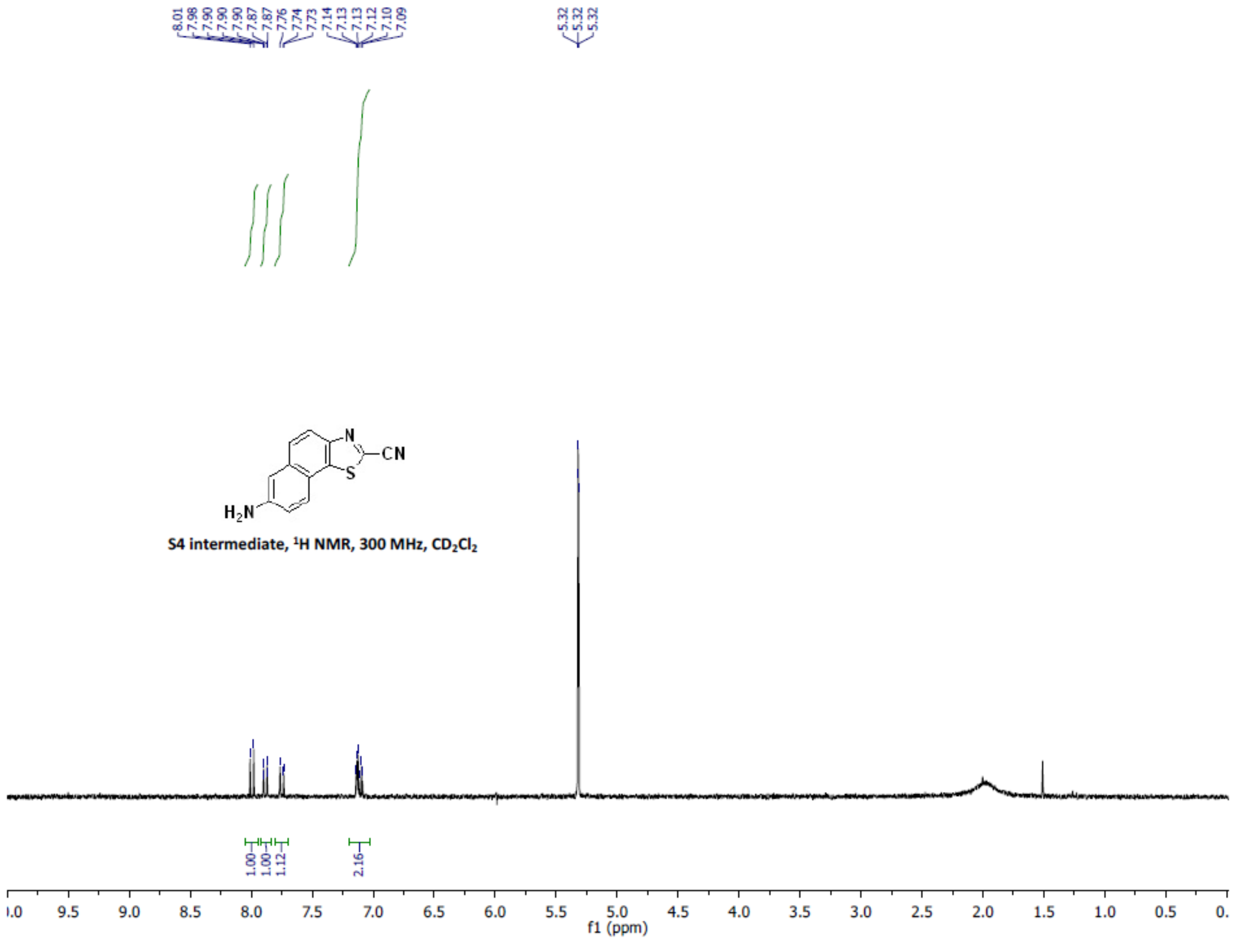
8.01
7.98
7.90
7.90
7.90
7.87
7.87
7.76
7.74
7.73
7.14
7.13
7.13
7.12
7.10
7.09
5.32
5.32
5.32
H2N
N
S
CN
S4 intermediate, ¹H NMR, 300 MHz, CD2Cl2
1.00
1.00
1.12
2.16
9.5
9.0
8.5
8.0
7.5
7.0
6.5
6.0
5.5
5.0
4.5
4.0
3.5
3.0
2.5
2.0
1.5
1.0
0.5
f1 (ppm)

(i)

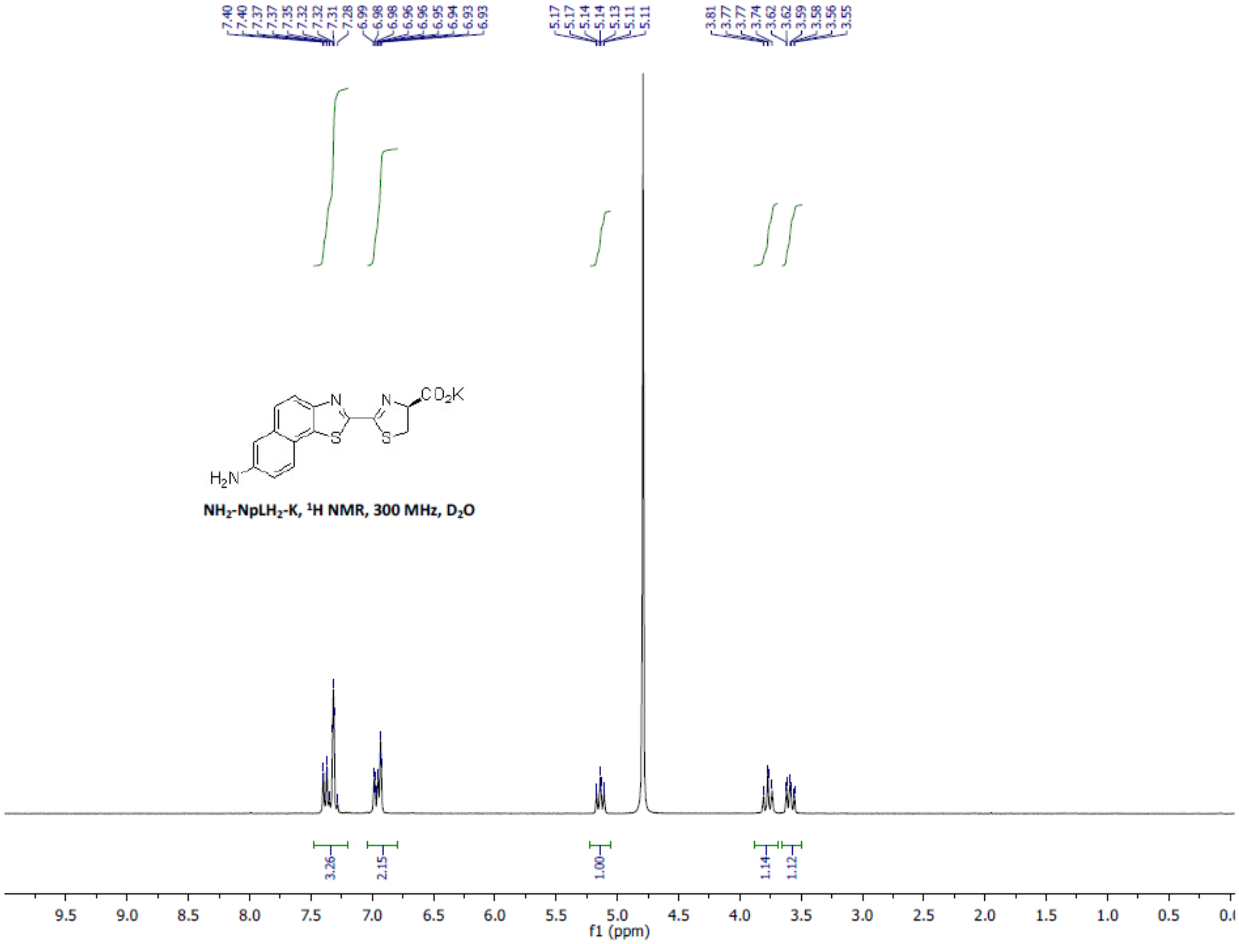

CO_2K
H_2N
NH_2-$NpLH_2$-K, ^1H NMR, 300 MHz, D_2O
3.26
2.15
1.00
1.14
1.12
9.5 9.0 8.5 8.0 7.5 7.0 6.5 6.0 5.5 5.0 4.5 4.0 3.5 3.0 2.5 2.0 1.5 1.0 0.5 0.[illegible]
f1 (ppm)

(j)

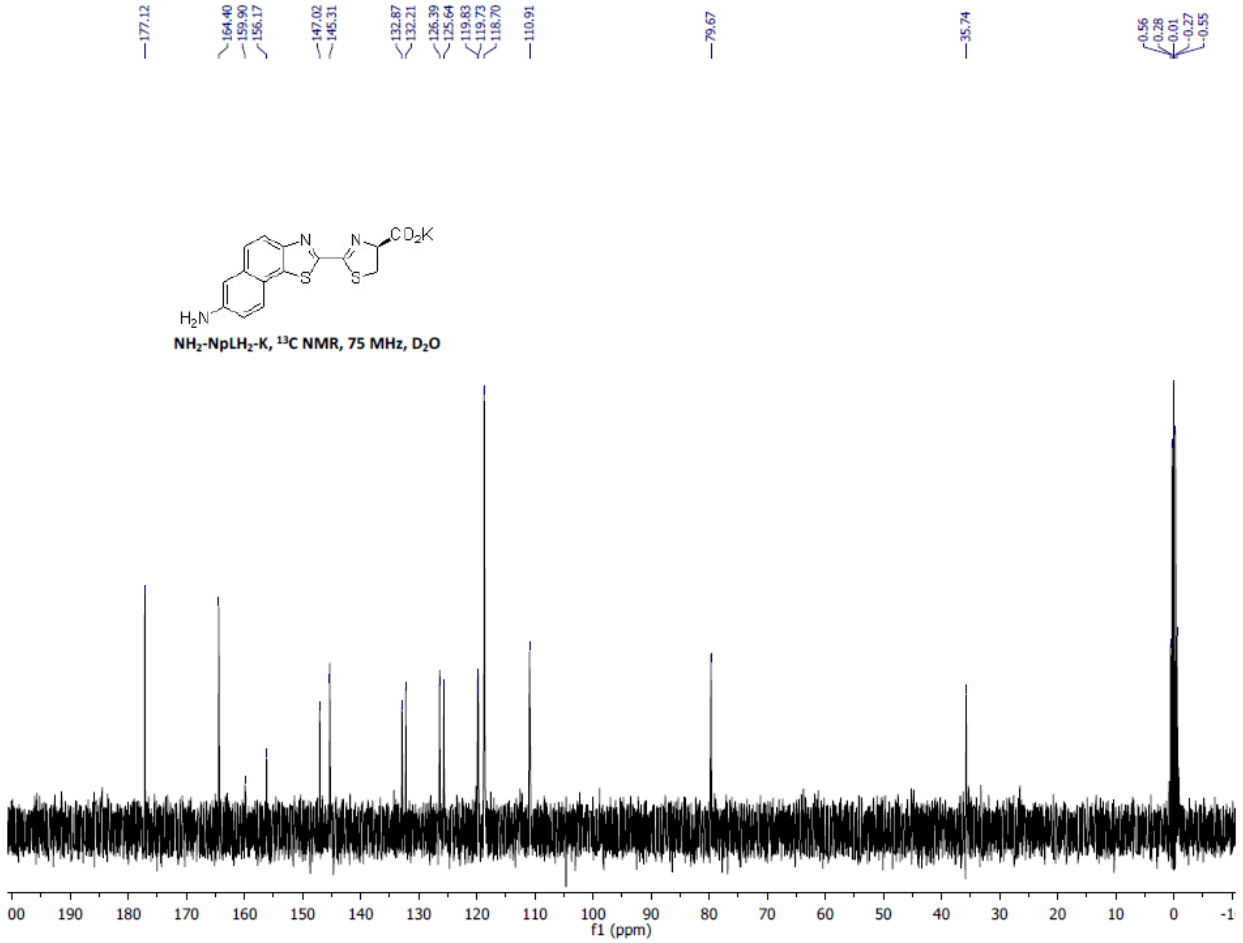

NH_2-NpLH_2-K, ^{13}C NMR, 75 MHz, D_2O

(k)

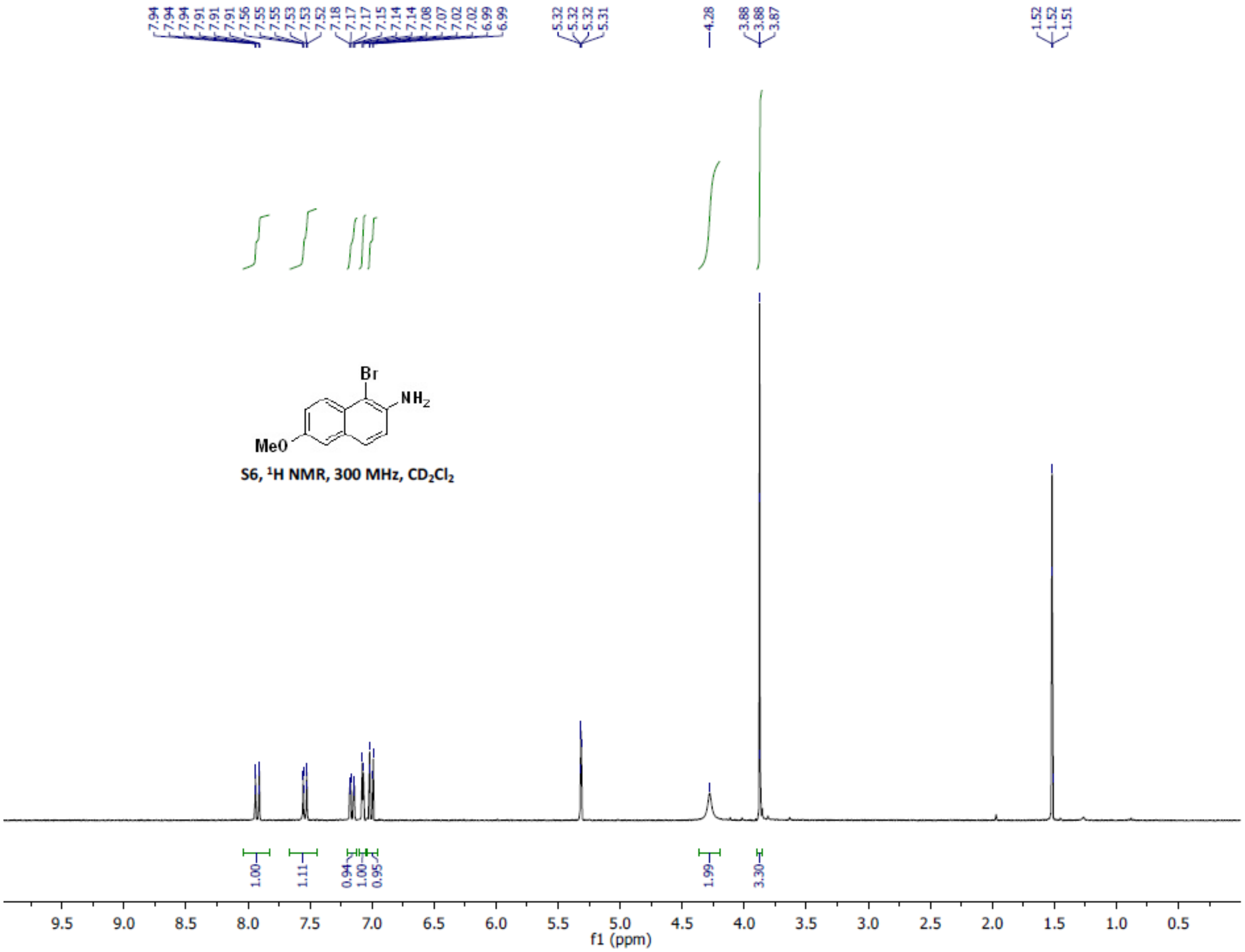

(l)

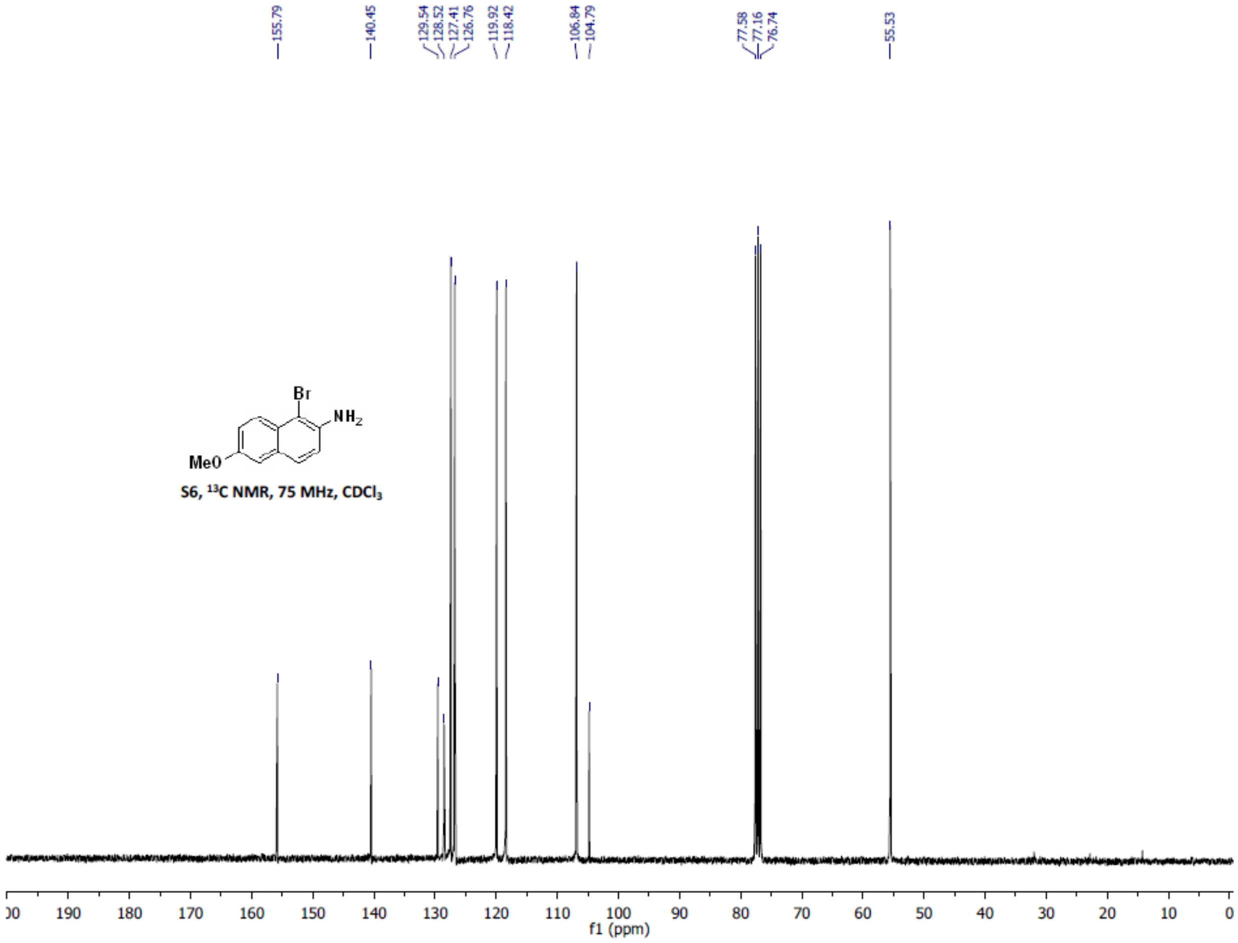

155.79
140.45
129.54
128.52
127.41
126.76
119.92
118.42
106.84
104.79
77.58
77.16
76.74
55.53
Br
NH2
MeO
S6, 13C NMR, 75 MHz, CDCl3
f1 (ppm)

(m)

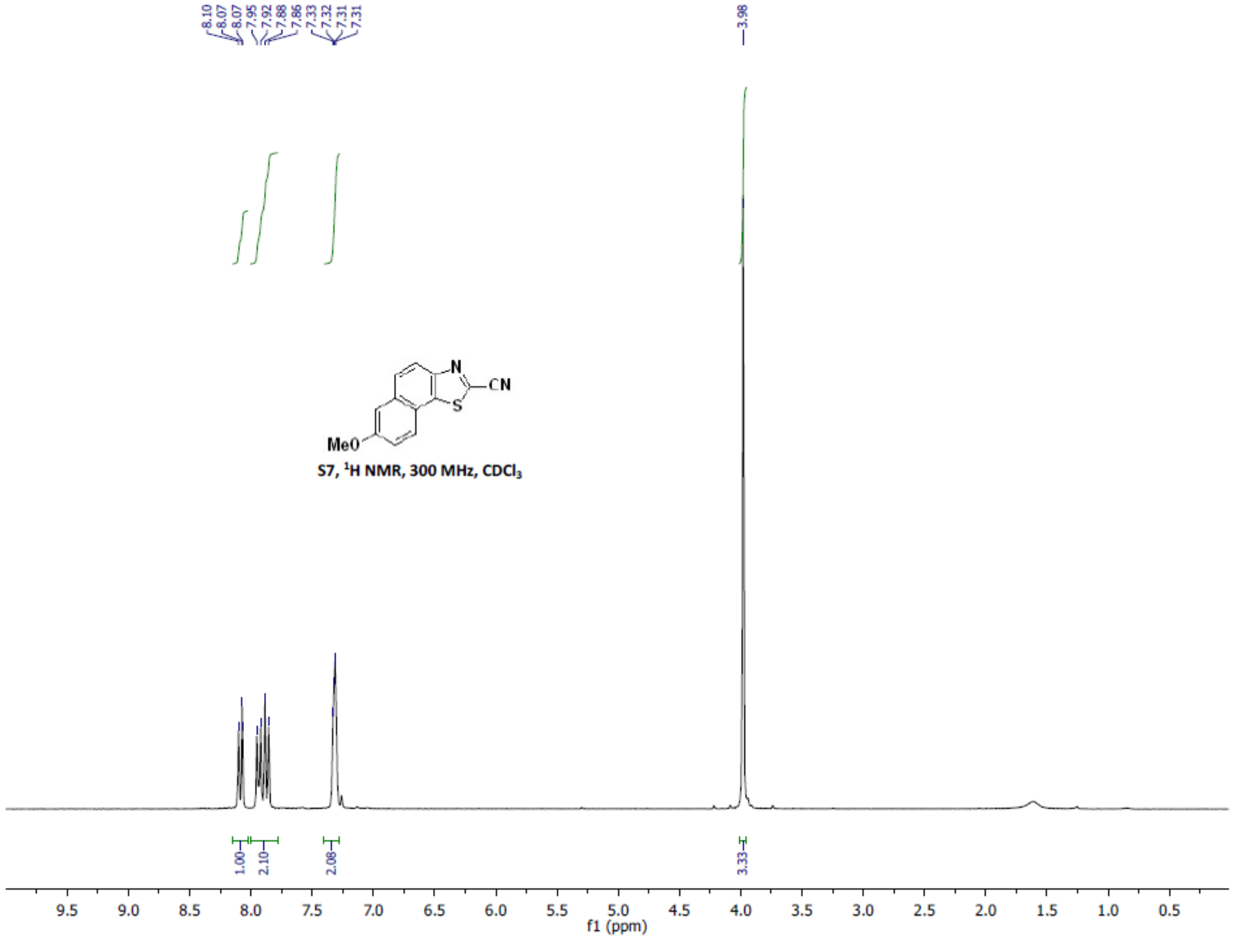

8.10
8.07
8.07
7.95
7.92
7.88
7.86
7.33
7.32
7.31
7.31
3.98
MeO
N
S
CN
S7, ¹H NMR, 300 MHz, CDCl₃
1.00
2.10
2.08
3.33
9.5
9.0
8.5
8.0
7.5
7.0
6.5
6.0
5.5
5.0
4.5
4.0
3.5
3.0
2.5
2.0
1.5
1.0
0.5
f1 (ppm)

(n)

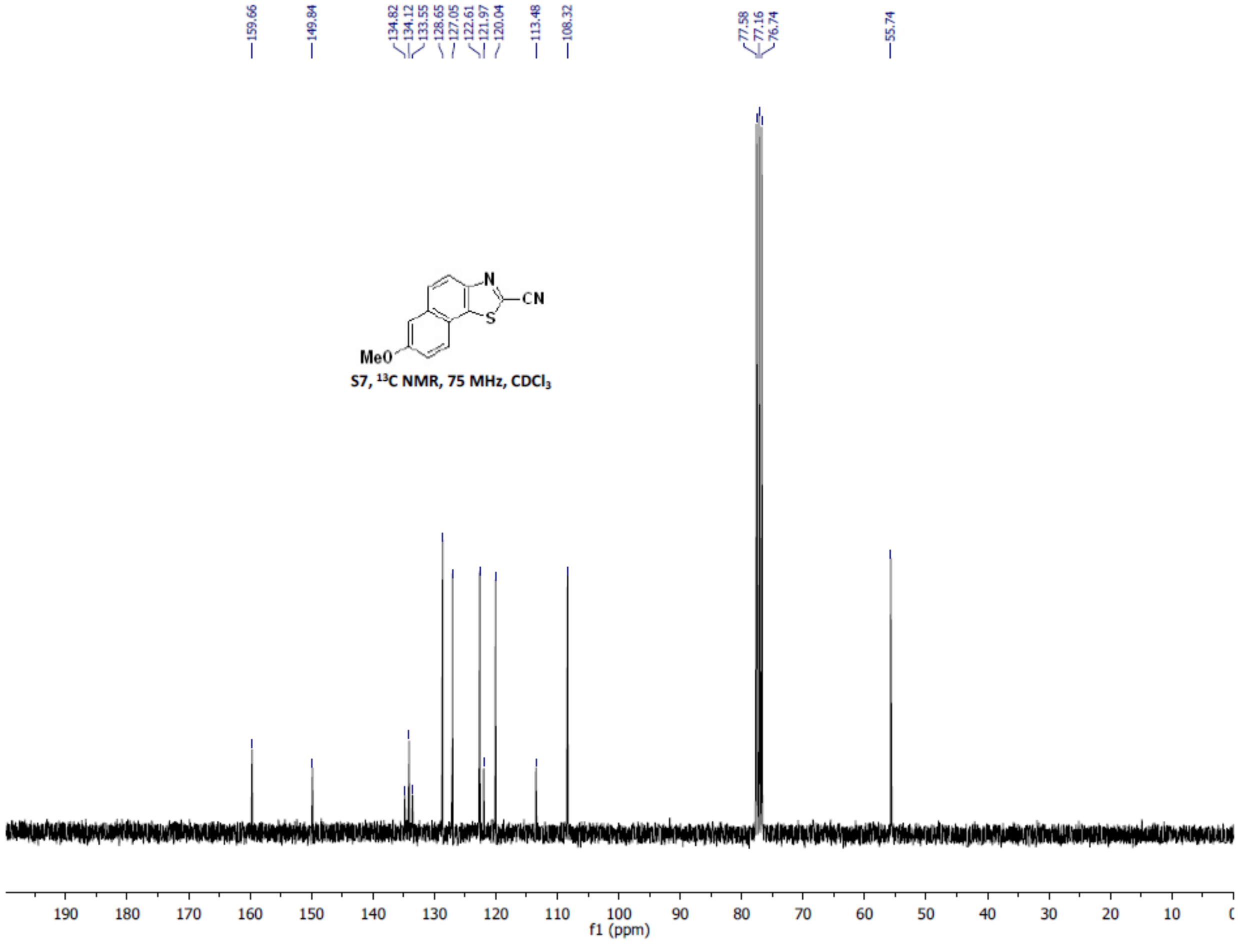

159.66
149.84
134.82
134.12
133.55
128.65
127.05
122.61
121.97
120.04
113.48
108.32
77.58
77.16
76.74
55.74
N
CN
S
MeO
S7, ¹³C NMR, 75 MHz, CDCl₃
190
180
170
160
150
140
130
120
110
100
90
80
70
60
50
40
30
20
10
f1 (ppm)

(o)

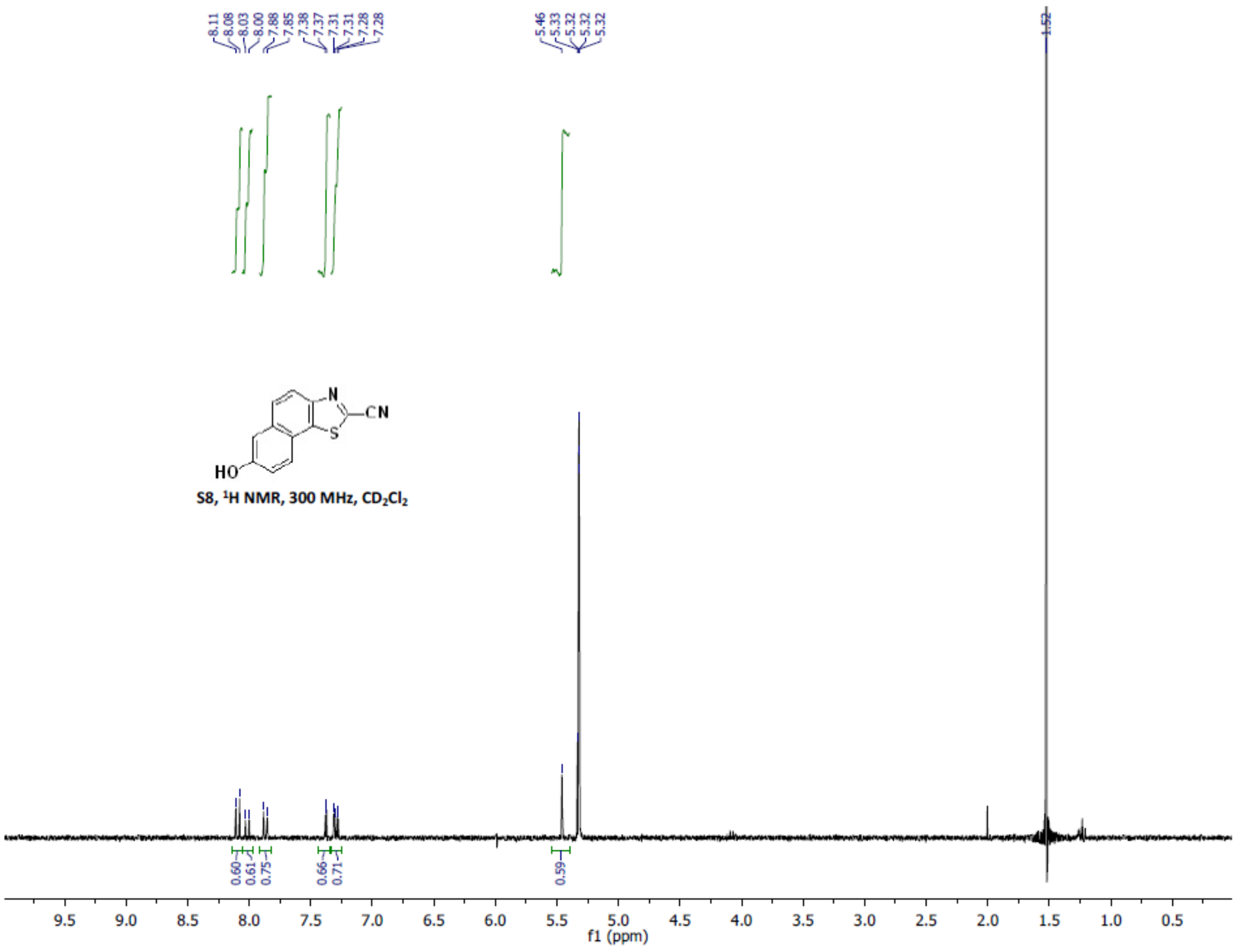
8.11
8.08
8.03
8.00
7.88
7.85
7.38
7.37
7.31
7.31
7.28
7.28
5.46
5.33
5.32
5.32
5.32
1.52
HO
N
S
CN
S8, ¹H NMR, 300 MHz, CD₂Cl₂
0.60
0.61
0.75
0.66
0.71
0.59
9.5
9.0
8.5
8.0
7.5
7.0
6.5
6.0
5.5
5.0
4.5
4.0
3.5
3.0
2.5
2.0
1.5
1.0
0.5
f1 (ppm)

(p)

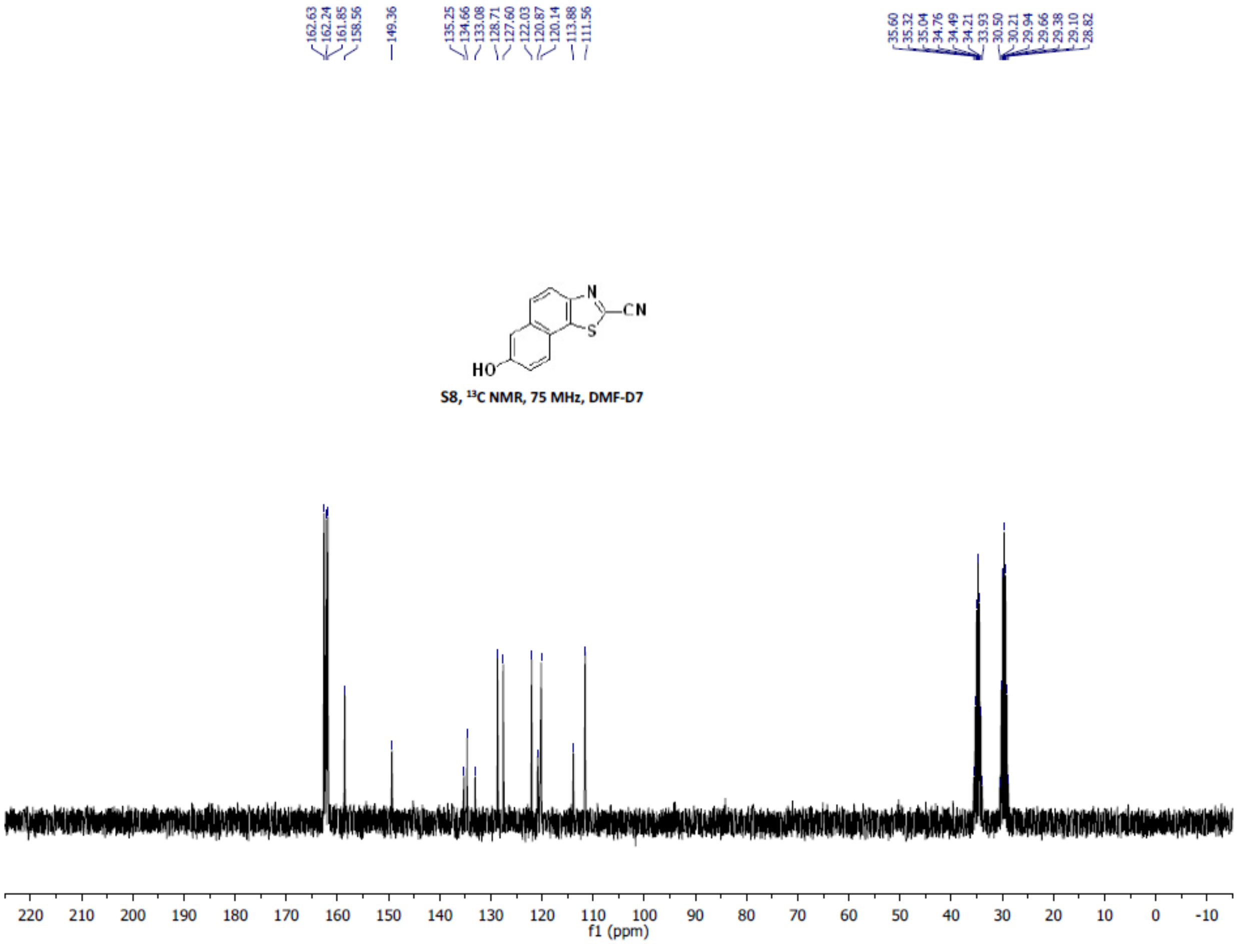

S8, ^{13}C NMR, 75 MHz, DMF-D7

(q)

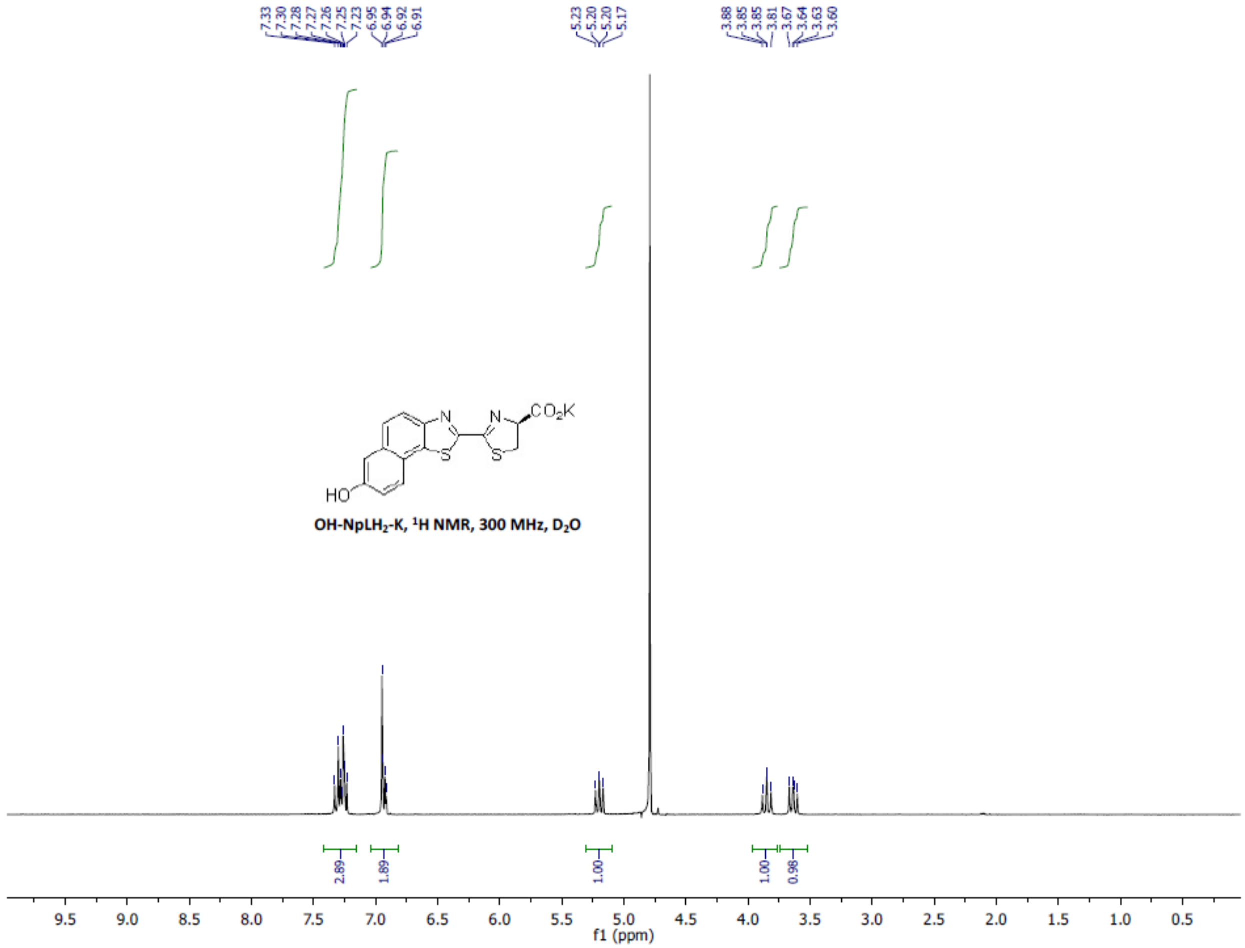

OH-NpLH$_2$-K, ^{1}H NMR, 300 MHz, D$_2$O

(r)

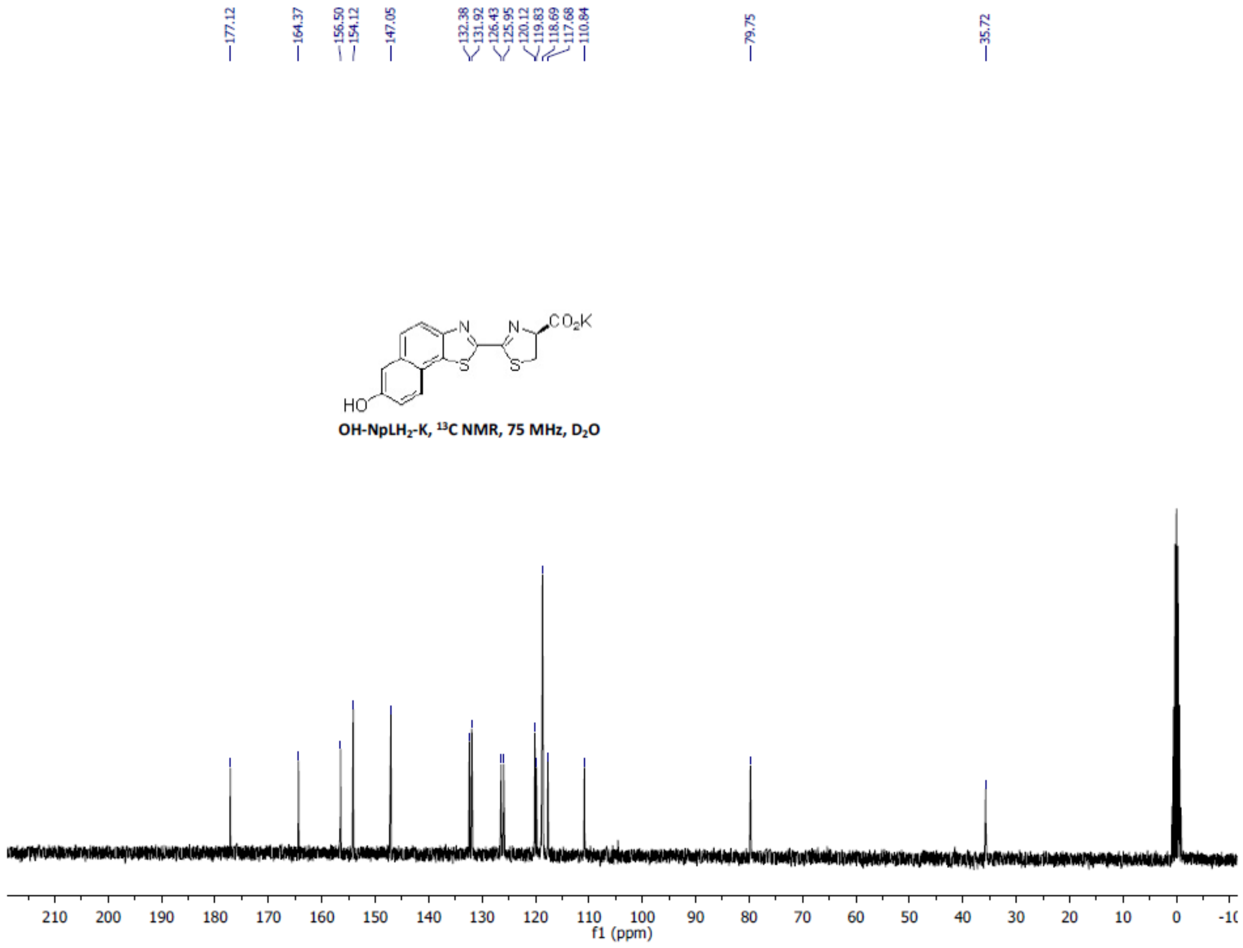

Supplementary Figure 3. NMR analysis. (**a**) ^{1}H NMR, 300 MHz, DMSO-D6, S2 intermediate. (**b**) ^{1}H NMR, 300 MHz, DMSO-D6, S2. (**c**) ^{13}C NMR, 75 MHz, $CDCl_3$, S2. (**d**) ^{1}H NMR, 300 MHz, CD_2Cl_2, S3. (**e**) ^{13}C NMR, 75 MHz, $CDCl_3$, S3. (**f**) ^{1}H NMR, 300 MHz, $CDCl_3$, S4. (**g**) ^{13}C NMR, 75 MHz, $CDCl_3$, S4. (**h**) ^{1}H NMR, 300 MHz, CD_2Cl_2, S4 intermediate. (**i**) ^{1}H NMR, 300 MHz, D_2O, NH_2-NpLH2-K. (**j**) ^{13}C NMR, 75 MHz, D_2O, NH_2-NpLH2-K. (**k**), ^{1}H NMR, 300 MHz, CD_2Cl_2, S6. (**l**) ^{13}C NMR, 75 MHz, $CDCl_3$, S6. (**m**) ^{1}H NMR, 300 MHz, $CDCl_3$, S7. (**n**) ^{13}C NMR, 75 MHz, $CDCl_3$, S7. (**o**) ^{1}H NMR, 300 MHz, CD_2Cl_2, S8. (**p**) ^{13}C NMR, 75 MHz, DMF-D7, S8. (**q**) ^{1}H NMR, 300 MHz, D_2O, OH-NpLH2-K. (**r**) ^{13}C NMR, 75 MHz, D_2O, OH-NpLH2-K.

```
AA Seq        1 M   V   K   R   E   K   N   V   I   Y   G   P   E   P   L     15
CBR2opt       1 ATG GTT AAG AGA GAA AAA AAT GTT ATT TAT GGT CCT GAA CCT CTG    45
                ||| ||. ||| .|. ||. ||| ||| ||. ||. ||| ||. ||| ||. ||| ||.
CBR2          1 ATG GTA AAG CGT GAG AAA AAT GTC ATC TAT GGC CCT GAG CCT CTC    45
AA Seq        1 M   V   K   R   E   K   N   V   I   Y   G   P   E   P   L     15

AA Seq       16 H   P   L   E   D   L   T   A   G   E   M   L   F   R   A     30
CBR2opt      46 CAT CCT CTG GAA GAT CTC ACT GCA GGA GAG ATG CTG TTT CGC GCC    90
                ||| ||| .|| ||. ||| .|. ||| ||. ||. ||. ||| ||| ||| ||. ||.
CBR2         46 CAT CCT TTG GAG GAT TTG ACT GCC GGC GAA ATG CTG TTT CGT GCT    90
AA Seq       16 H   P   L   E   D   L   T   A   G   E   M   L   F   R   A     30

AA Seq       31 L   R   K   H   S   H   L   P   Q   A   L   V   D   V   V     45
CBR2opt      91 CTG CGC AAG CAC TCT CAC TTG CCG CAG GCT CTG GTC GAC GTG GTG   135
                ||. ||| ||| ||| ||| ||. ||| ||. ||. ||. .|| ||| ||. ||| ||.
CBR2         91 CTC CGC AAG CAC TCT CAT TTG CCT CAA GCC TTG GTC GAT GTG GTC   135
AA Seq       31 L   R   K   H   S   H   L   P   Q   A   L   V   D   V   V     45

AA Seq       46 G   D   E   S   L   S   Y   K   E   F   F   E   A   T   V     60
CBR2opt     136 GGT GAC GAG TCC CTG AGC TAC AAG GAG TTC TTC GAG GCG ACT GTC   180
                ||. ||. ||. ||. .|| ||| ||| ||| ||| ||. ||. ||| ||. ||. |||
CBR2        136 GGC GAT GAA TCT TTG AGC TAC AAG GAG TTT TTT GAG GCA ACC GTC   180
AA Seq       46 G   D   E   S   L   S   Y   K   E   F   F   E   A   T   V     60

AA Seq       61 L   L   A   Q   S   L   H   N   C   G   Y   K   M   N   D     75
CBR2opt     181 CTG CTG GCA CAG AGT CTC CAT AAC TGC GGC TAC AAG ATG AAC GAT   225
                .|| ||| ||. ||| ... ||| ||. ||. ||. ||| ||| ||| ||| ||| ||.
CBR2        181 TTG CTG GCT CAG TCC CTC CAC AAT TGT GGC TAC AAG ATG AAC GAC   225
AA Seq       61 L   L   A   Q   S   L   H   N   C   G   Y   K   M   N   D     75

AA Seq       76 V   V   S   I   C   A   E   N   N   T   R   F   F   I   P     90
CBR2opt     226 GTG GTT TCC ATC TGC GCA GAG AAC AAC ACT AGA TTC TTT ATC CCA   270
                ||. ||| ... ||| ||. ||. ||. ||| ||. ||. .|. ||| ||. ||. |||
CBR2        226 GTC GTT AGT ATC TGT GCT GAA AAC AAT ACC CGT TTC TTC ATT CCA   270
AA Seq       76 V   V   S   I   C   A   E   N   N   T   R   F   F   I   P     90

AA Seq       91 V   I   A   A   W   Y   I   G   M   I   V   A   P   V   N    105
CBR2opt     271 GTG ATC GCT GCT TGG TAC ATC GGC ATG ATT GTC GCA CCA GTC AAT   315
                ||. ||| ||. ||. ||| ||. ||| ||. ||| ||. ||. ||. ||| ||| ||.
CBR2        271 GTC ATC GCC GCA TGG TAT ATC GGT ATG ATC GTG GCT CCA GTC AAC   315
AA Seq       91 V   I   A   A   W   Y   I   G   M   I   V   A   P   V   N    105

AA Seq      106 E   S   Y   I   P   D   E   L   C   K   V   M   G   I   S    120
CBR2opt     316 GAA TCT TAC ATT CCA GAT GAA CTG TGT AAG GTT ATG GGA ATT TCC   360
                ||. ... ||| ||| ||. ||. ||| ||| ||| ||. ||. ||| ||. ||. ||.
CBR2        316 GAG AGC TAC ATT CCC GAC GAA CTG TGT AAA GTC ATG GGT ATC TCT   360
AA Seq      106 E   S   Y   I   P   D   E   L   C   K   V   M   G   I   S    120

AA Seq      121 K   P   Q   I   V   F   T   T   K   N   I   L   N   K   V    135
CBR2opt     361 AAA CCG CAG ATC GTG TTC ACA ACT AAG AAC ATC CTG AAC AAG GTT   405
                ||. ||. ||| ||. ||. ||| ||. ||| ||| ||. ||. ||| ||| ||. ||.
CBR2        361 AAG CCA CAG ATT GTC TTC ACC ACT AAG AAT ATT CTG AAC AAA GTC   405
AA Seq      121 K   P   Q   I   V   F   T   T   K   N   I   L   N   K   V    135

AA Seq      136 L   E   V   Q   S   R   T   N   F   I   K   R   I   I   I    150
CBR2opt     406 CTG GAA GTG CAG AGT CGG ACC AAT TTC ATC AAG CGT ATT ATC ATC   450
                ||| ||| ||. ||. ||. ||. ||| ||. ||. ||. ||| ||| ||. ||| |||
CBR2        406 CTG GAA GTC CAA AGC CGC ACC AAC TTT ATT AAG CGT ATC ATC ATC   450
AA Seq      136 L   E   V   Q   S   R   T   N   F   I   K   R   I   I   I    150
```

```
AA Seq          151 L   D   T   V   E   N   I   H   G   C   E   S   L   P   N      165
CBR2opt         451 CTG GAC ACA GTG GAG AAC ATC CAC GGT TGC GAA TCC CTC CCC AAC    495
                    .|| ||| ||. ||| ||| ||. ||. ||| ||| ||| ||| ||. .|. ||. ||.
CBR2            451 TTG GAC ACT GTG GAG AAT ATT CAC GGT TGC GAA TCT TTG CCT AAT    495
AA Seq          151 L   D   T   V   E   N   I   H   G   C   E   S   L   P   N      165

AA Seq          166 F   I   S   R   Y   S   D   G   N   I   A   N   F   K   P      180
CBR2opt         496 TTC ATC TCT CGT TAT AGC GAT GGC AAC ATC GCT AAT TTC AAA CCC    540
                    ||| ||| ||| ||. ||| ... ||. ||| ||| ||| ||. ||. ||. ||| ||.
CBR2            496 TTC ATC TCT CGC TAT TCA GAC GGC AAC ATC GCA AAC TTT AAA CCA    540
AA Seq          166 F   I   S   R   Y   S   D   G   N   I   A   N   F   K   P      180

AA Seq          181 L   H   F   D   P   V   E   Q   V   A   A   I   L   C   S      195
CBR2opt         541 CTG CAT TTC GAT CCT GTT GAA CAG GTT GCC GCC ATC CTG TGC TCC    585
                    ||. ||. ||| ||. ||| ||. ||| ||. ||| ||. ||| ||. ||| ||. ..|
CBR2            541 CTC CAC TTC GAC CCT GTG GAA CAA GTT GCA GCC ATT CTG TGT AGC    585
AA Seq          181 L   H   F   D   P   V   E   Q   V   A   A   I   L   C   S      195

AA Seq          196 S   G   T   T   G   L   P   K   G   V   M   Q   T   H   Q      210
CBR2opt         586 TCT GGG ACC ACC GGA CTG CCG AAA GGC GTG ATG CAG ACC CAT CAG    630
                    ... ||. ||. ||. ||| ||. ||. ||. ||. ||. ||| ||| ||| ||| ||.
CBR2            586 AGC GGT ACT ACT GGA CTC CCA AAG GGA GTC ATG CAG ACC CAT CAA    630
AA Seq          196 S   G   T   T   G   L   P   K   G   V   M   Q   T   H   Q      210

AA Seq          211 N   I   C   V   R   L   I   H   A   L   D   P   R   Y   G      225
CBR2opt         631 AAC ATC TGC GTG AGG CTG ATC CAC GCC CTG GAC CCA CGC TAT GGA    675
                    ||| ||. ||| ||| .|. ||| ||| ||. ||. ||. ||. ||| ||| ||. ||.
CBR2            631 AAC ATT TGC GTG CGT CTG ATC CAT GCT CTC GAT CCA CGC TAC GGC    675
AA Seq          211 N   I   C   V   R   L   I   H   A   L   D   P   R   Y   G      225

AA Seq          226 T   Q   L   I   P   G   V   T   V   L   V   Y   L   P   F      240
CBR2opt         676 ACT CAG CTG ATC CCA GGA GTC ACT GTG CTG GTT TAC CTC CCT TTT    720
                    ||| ||| ||| ||. ||. ||. ||| ||. ||. .|| ||. ||| .|. ||| ||.
CBR2            676 ACT CAG CTG ATT CCT GGT GTC ACC GTC TTG GTC TAC TTG CCT TTC    720
AA Seq          226 T   Q   L   I   P   G   V   T   V   L   V   Y   L   P   F      240

AA Seq          241 F   H   A   F   G   F   H   I   T   L   G   Y   F   M   V      255
CBR2opt         721 TTT CAC GCC TTC GGT TTC CAC ATT ACC CTC GGG TAC TTT ATG GTC    765
                    ||. ||. ||. ||| ||. ||. ||. ||| ||. .|. ||. ||| ||| ||| |||
CBR2            721 TTC CAT GCT TTC GGC TTT CAT ATT ACT TTG GGT TAC TTT ATG GTC    765
AA Seq          241 F   H   A   F   G   F   H   I   T   L   G   Y   F   M   V      255

AA Seq          256 G   L   R   V   I   M   F   R   R   F   D   Q   E   A   F      270
CBR2opt         766 GGC CTC CGC GTT ATC ATG TTC CGT CGC TTT GAC CAG GAG GCT TTC    810
                    ||. ||| ||| ||. ||. ||| ||| ||. ||. ||| ||. ||| ||| ||| |||
CBR2            766 GGT CTC CGC GTG ATT ATG TTC CGC CGT TTT GAT CAG GAG GCT TTC    810
AA Seq          256 G   L   R   V   I   M   F   R   R   F   D   Q   E   A   F      270

AA Seq          271 L   K   A   I   Q   D   Y   E   V   R   S   V   I   N   V      285
CBR2opt         811 CTG AAG GCC ATC CAG GAT TAT GAG GTG AGG AGT GTG ATT AAC GTC    855
                    .|| ||. ||| ||| ||. ||| ||| ||. ||. .|. ||| ||. ||. ||| ||.
CBR2            811 TTG AAA GCC ATC CAA GAT TAT GAA GTC CGC AGT GTC ATC AAC GTG    855
AA Seq          271 L   K   A   I   Q   D   Y   E   V   R   S   V   I   N   V      285

AA Seq          286 P   S   V   I   L   F   L   S   K   S   P   L   V   D   K      300
CBR2opt         856 CCA AGC GTG ATC CTG TTC CTG TCT AAG TCC CCT CTG GTT GAC AAG    900
                    ||. ||| ||| ||| ||| ||. .|| ||| ||| ..| ||. ||. ||. ||| |||
CBR2            856 CCT AGC GTG ATC CTG TTT TTG TCT AAG AGC CCA CTC GTG GAC AAG    900
AA Seq          286 P   S   V   I   L   F   L   S   K   S   P   L   V   D   K      300
```

```
                                           334
AA Seq    301  Y   D   L   S   S   L   R   E   L   C   C   G   A   A   P    315
CBR2opt   901 TAC GAT CTG AGC TCC CTG CGT GAG CTG TGC TGC GGG GCT GCA CCA   945
              ||| ||. .|| ... ||. ||| ||| ||. .|| ||. ||| ||. ||. ||. |||
CBR2      901 TAC GAC TTG TCT TCA CTG CGT GAA TTG TGT TGC GGT GCC GCT CCA   945
AA Seq    301  Y   D   L   S   S   L   R   E   L   C   C   G   A   A   P    315


AA Seq    316  L   A   K   E   V   A   E   V   A   A   K   R   L   N   L    330
CBR2opt   946 CTG GCC AAA GAA GTC GCG GAA GTG GCT GCC AAA CGC CTG AAC CTG   990
              ||| ||. ||. ||. ||| ||. ||| ||| ||. ||| ||| ||| .|| ||. ||.
CBR2      946 CTG GCT AAG GAG GTC GCT GAA GTG GCC GCC AAA CGC TTG AAT CTT   990
AA Seq    316  L   A   K   E   V   A   E   V   A   A   K   R   L   N   L    330

                          334
AA Seq    331  P   G   I   S   C   G   F   G   L   T   E   S   T   S   A    345
CBR2opt   991 CCG GGT ATC TCC TGT GGC TTC GGC CTG ACA GAG AGC ACA TCC GCT  1035
              ||. ||. ||. ... ||| ||| ||| ||| ||. ||. ||. ... ||. ... ||.
CBR2      991 CCA GGG ATT AGT TGT GGC TTC GGC CTC ACC GAA TCT ACC AGT GCG  1035
AA Seq    331  P   G   I   S   C   G   F   G   L   T   E   S   T   S   A    345

                                  351
AA Seq    346  I   I   Q   T   L   R   D   E   F   K   S   G   S   L   G    360
CBR2opt  1036 ATC ATC CAG ACA CTG CGG GAT GAG TTT AAG TCC GGT TCC CTG GGC  1080
              ||. ||| ||| ||. ||. ||| ||| ||| ||| ||| ..| ||. ||. .|| |||
CBR2     1036 ATT ATC CAG ACT CTC CGG GAT GAG TTT AAG AGC GGC TCT TTG GGC  1080
AA Seq    346  I   I   Q   T   L   R   D   E   F   K   S   G   S   L   G    360


AA Seq    361  R   V   T   P   L   M   A   A   K   I   A   D   R   E   T    375
CBR2opt  1081 CGG GTT ACA CCC CTG ATG GCA GCC AAG ATC GCC GAC CGC GAG ACT  1125
              ||. ||. ||. ||. ||. ||| ||. ||. ||| ||| ||. ||. ||| ||. |||
CBR2     1081 CGT GTC ACT CCA CTC ATG GCT GCT AAG ATC GCT GAT CGC GAA ACT  1125
AA Seq    361  R   V   T   P   L   M   A   A   K   I   A   D   R   E   T    375


AA Seq    376  G   K   A   L   G   P   N   Q   V   G   E   L   C   I   K    390
CBR2opt  1126 GGG AAG GCC CTC GGA CCT AAC CAG GTC GGA GAG CTG TGT ATT AAG  1170
              ||. ||| ||. .|. ||. ||. ||| ||. ||. ||. ||| ||| ||| ||. ||.
CBR2     1126 GGT AAG GCT TTG GGC CCG AAC CAA GTG GGC GAG CTG TGT ATC AAA  1170
AA Seq    376  G   K   A   L   G   P   N   Q   V   G   E   L   C   I   K    390


AA Seq    391  G   P   M   V   S   K   G   Y   V   N   N   V   E   A   T    405
CBR2opt  1171 GGC CCC ATG GTG TCC AAG GGA TAT GTG AAC AAC GTG GAA GCT ACA  1215
              ||| ||. ||| ||| ..| ||| ||. ||| ||. ||. ||| ||. ||| ||| ||.
CBR2     1171 GGC CCT ATG GTG AGC AAG GGT TAT GTC AAT AAC GTT GAA GCT ACC  1215
AA Seq    391  G   P   M   V   S   K   G   Y   V   N   N   V   E   A   T    405


AA Seq    406  K   E   A   I   D   D   D   G   W   L   H   S   G   D   F    420
CBR2opt  1216 AAG GAG GCG ATT GAT GAT GAC GGA TGG CTC CAT AGC GGA GAC TTT  1260
              ||| ||| ||. ||. ||. ||. ||| ||. ||| .|. ||| ... ||. ||. |||
CBR2     1216 AAG GAG GCC ATC GAC GAC GAC GGC TGG TTG CAT TCT GGT GAT TTT  1260
AA Seq    406  K   E   A   I   D   D   D   G   W   L   H   S   G   D   F    420


AA Seq    421  G   Y   Y   D   E   D   E   H   F   Y   V   V   D   R   Y    435
CBR2opt  1261 GGG TAC TAT GAC GAG GAC GAG CAC TTC TAT GTG GTC GAC AGG TAC  1305
              ||. ||. ||. ||| ||. ||. ||| ||. ||. ||. ||. ||. ||. .|. |||
CBR2     1261 GGA TAT TAC GAC GAA GAT GAG CAT TTT TAC GTC GTG GAT CGT TAC  1305
AA Seq    421  G   Y   Y   D   E   D   E   H   F   Y   V   V   D   R   Y    435


AA Seq    436  K   E   L   I   K   Y   K   G   S   Q   V   A   P   A   E    450
CBR2opt  1306 AAG GAG CTG ATC AAG TAC AAG GGC TCC CAG GTG GCC CCA GCT GAA  1350
              ||| ||| ||| ||| ||. ||| ||| ||. ..| ||| ||. ||. ||| ||| ||.
CBR2     1306 AAG GAG CTG ATC AAA TAC AAG GGT AGC CAG GTT GCT CCA GCT GAG  1350
AA Seq    436  K   E   L   I   K   Y   K   G   S   Q   V   A   P   A   E    450
```

```
AA Seq          451  L   E   E   I   L   L   K   N   P   C   I   R   D   V   A     465
CBR2opt        1351 CTG GAG GAG ATT CTG TTG AAG AAC CCT TGT ATC AGA GAT GTG GCT   1395
                    .|| ||| ||| ||| ||| ||| ||. ||. ||. ||. ||. .|. ||| ||. |||
CBR2           1351 TTG GAG GAG ATT CTG TTG AAA AAT CCA TGC ATT CGC GAT GTC GCT   1395
AA Seq          451  L   E   E   I   L   L   K   N   P   C   I   R   D   V   A     465

AA Seq          466  V   V   G   I   P   D   L   E   A   G   E   L   P   S   A     480
CBR2opt        1396 GTT GTG GGG ATT CCT GAC CTC GAA GCA GGT GAG CTG CCC TCC GCT   1440
                    ||. ||. ||. ||| ||| ||. ||. ||. ||. ||. ||. ||| ||. ||. |||
CBR2           1396 GTG GTC GGC ATT CCT GAT CTG GAG GCC GGC GAA CTG CCT TCT GCT   1440
AA Seq          466  V   V   G   I   P   D   L   E   A   G   E   L   P   S   A     480

AA Seq          481  F   V   V   K   Q   P   G   T   E   I   T   A   K   E   V     495
CBR2opt        1441 TTC GTG GTG AAG CAG CCT GGT ACT GAG ATC ACC GCC AAA GAG GTG   1485
                    ||| ||. ||. ||| ||| ||| ||| ||. ||. ||. ||| ||| ||| ||. |||
CBR2           1441 TTC GTT GTC AAG CAG CCT GGT ACA GAA ATT ACC GCC AAA GAA GTG   1485
AA Seq          481  F   V   V   K   Q   P   G   T   E   I   T   A   K   E   V     495

AA Seq          496  Y   D   Y   L   A   E   R   V   S   H   T   K   Y   L   R     510
CBR2opt        1486 TAC GAT TAC CTG GCT GAG CGC GTG TCC CAC ACC AAA TAC CTC CGC   1530
                    ||. ||| ||| ||| ||| ||. ||. ||| ..| ||. ||. ||. ||| .|. ||.
CBR2           1486 TAT GAT TAC CTG GCT GAA CGT GTG AGC CAT ACT AAG TAC TTG CGT   1530
AA Seq          496  Y   D   Y   L   A   E   R   V   S   H   T   K   Y   L   R     510

AA Seq          511  G   G   V   R   F   V   D   S   I   P   R   N   V   T   G     525
CBR2opt        1531 GGC GGG GTC CGT TTC GTG GAC TCC ATT CCC AGG AAC GTG ACC GGC   1575
                    ||| ||. ||. ||| ||. ||. ||| ||| ||. ||. .|. ||| ||. ||. |||
CBR2           1531 GGC GGC GTG CGT TTT GTT GAC TCC ATC CCT CGT AAC GTA ACA GGC   1575
AA Seq          511  G   G   V   R   F   V   D   S   I   P   R   N   V   T   G     525

AA Seq          526  K   I   T   R   K   E   L   L   K   Q   L   L   V   K   A     540
CBR2opt        1576 AAG ATC ACC CGC AAG GAG CTG CTG AAG CAG CTG CTG GTT AAG GCT   1620
                    ||. ||. ||| ||| ||| ||| ||| .|| ||. ||. .|| .|| ||. ||| ||.
CBR2           1576 AAA ATT ACC CGC AAG GAG CTG TTG AAA CAA TTG TTG GTG AAG GCC   1620
AA Seq          526  K   I   T   R   K   E   L   L   K   Q   L   L   V   K   A     540

AA Seq          541  G   G   V   *      544
CBR2opt        1621 GGC GGC GTT TAA    1632
                    ||| ||. ||| |||
CBR2           1621 GGC GGT GTT TAA    1632
AA Seq          541  G   G   V   *      544
```

Supplementary Fig. 4. Alignment of CBR2opt and CBR2 (1,632 nucleotides). The two DNA sequences utilize different codons but encode the same enzyme sequence. CBR2opt and CBR2 share 78.6% sequence identity.

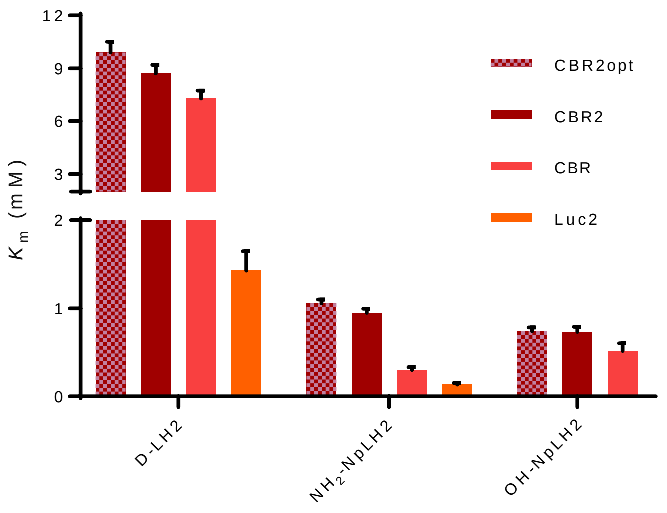

Supplementary Figure 5. Calculated K_m values for D-LH2, NH_2-NpLH2, and OH-NpLH2 in HEK-293 cells transiently expressing Luc2, CBR, CBR2, or CBR2opt (data presented as means (n=3) ± standard deviation (S.D.)). Error bars = S.D.

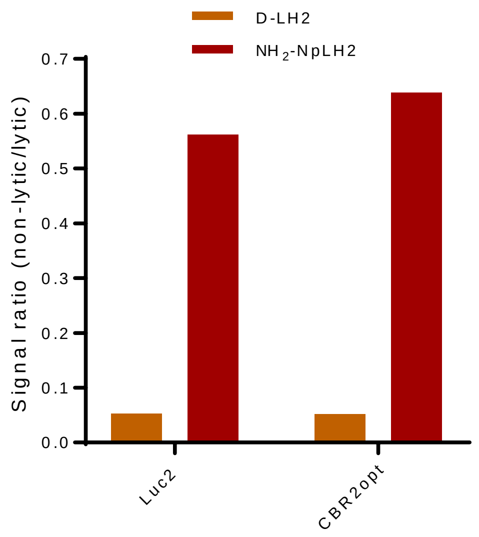

Supplementary Figure 6. Membrane permeability of D-LH2 and NH_2-NpLH2 in HEK-293 cells transiently expressing Luc2 or CBR2opt. Data represents ratios of the signals produced from non-lytic conditions to lytic conditions for D-LH2 and NH_2-NpLH2 (as produced by either Luc2 or CBR2opt).

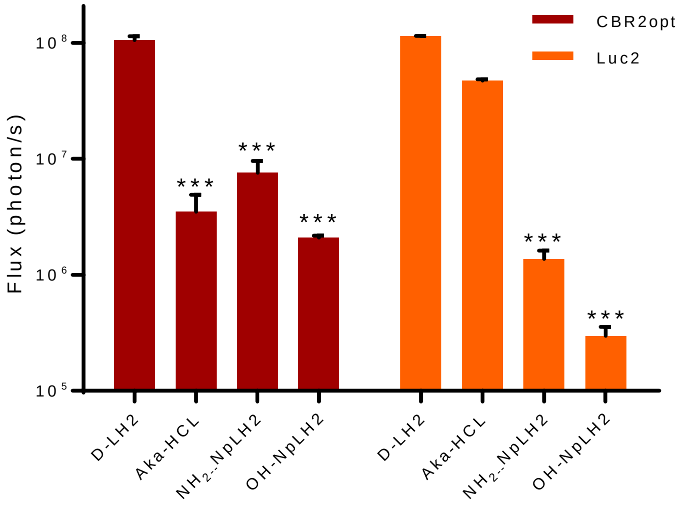

Supplementary Figure 7. Bioluminescence emission (photon flux; CCD camera) for D-LH2, NH_2-NpLH2, OH-NpLH2, and Aka-HCl produced by Luc2 or CBR2opt expressed stably in MCF-7 cells; 10 min time point. Each column is compared to CBR2opt/D-LH2 or Luc2/D-LH2 (***, p<0.001; ONE-Way ANOVA followed by Tukey's T test; data presented as means (n=3) ± standard deviation (S.D.)). Error bars = S.D.

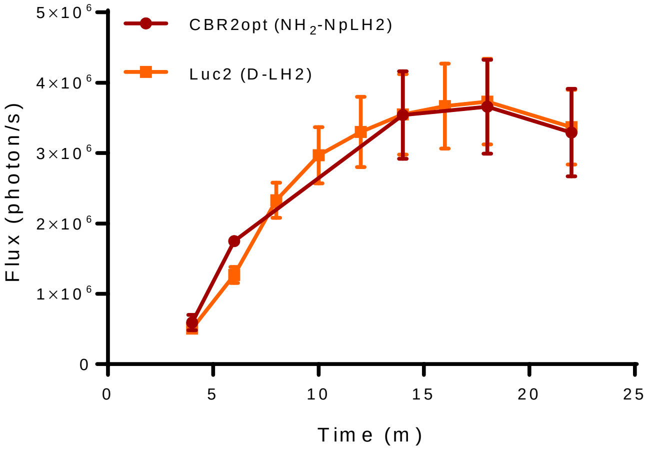

Supplementary Figure 8. Relatively poor kinetics for bioluminescence generation in mouse brain following intraperitoneal administration of substrate (data presented as means (n=3) ± standard deviation (S.D.)). Error bars = S.D.

Supplementary Table 1. Chiral HPLC conditions.

Time (min)	%H_2O (0.1% TFA)	%CH_3CN	Flow rate (mL min^{-1})
0	75	25	0.6
17	55	45	0.6
19	20	80	0.6
24	20	80	0.6
25	75	25	0.6
30	75	25	0.6

Supplementary Methods

Synthesis of NIR naphthyl-luciferin substrates

All reagents and solvents for chemical syntheses were purchased from Sigma-Aldrich or Thermo Fisher Scientific and were used without further purification. The purity of the substrates was assessed on an analytical HPLC (Agilent 1100 Series) by monitoring absorbance at 254 nm. The enantiopurity was analyzed on an analytical HPLC (Agilent 1100 Series) using Chiralcel® OD-RH (150 X 4.6 mm ID). Nuclear Magnetic Resonance (NMR) and mass spectra were recorded on a Varian 300 and a Waters LCMS instrument with Waters 2695 Separation Module/3100 Mass Detector. Waters Preparative HPLC (Waters 2487 Series) was used to purify the products by using 10 mM aqueous NH_4OAc and MeCN as eluents.

***tert*-Butyl (6-aminonaphthalen-2-yl)carbamate (S2).** 2-(*tert*-Butylcarbamoyl)amino-6-naphthoic acid (**S1**, 1.35 g, 4.7 mmol) was stirred in 20 mL of DCM in an ice bath. DMF (0.2 mL) was added, and then oxalyl chloride (420 µL, 630 mg, 4.9 mmol) added slowly. After 45 min, the reaction was concentrated under reduced pressure, and the residue was taken up in acetone (20 mL). Sodium azide (916 mg, 14.1 mmol) was added as a solution in H_2O (15 mL), and the reaction was stirred for 15 min. The desired intermediate, *tert*-butyl (6-(azidocarbonyl)naphthalen-2-yl)carbamate, was precipitated by the addition of H_2O (100 mL) and isolated by filtration as slightly pink solid (1.26 g, 86% isolated yield). ^{1}H NMR (300 MHz, DMSO-d_6) δ 9.81 (br s, 1H); 8.51 (s, 1H); 8.21 (d, J = 2.0 Hz, 1H); 8.06 (d, J = 9.0 Hz, 1H); 7.84-7.91 (m, 2H); 7.61 (dd, J = 9.0, 2.0 Hz, 1H); 1.50 (s, 9H); LC-MS (ESI) m/z = 313.2 $[M + H]^+$.

The intermediate from previous step (2.7 g, 8.6 mmol) in toluene (75 mL) was heated to reflux for 2 h. To the solution, was added 6 N NaOH aqueous solution (10 mL, 60 mmol). The mixture was heated at reflux for 72 h. The reaction was cooled to ambient temperature and filtered. The filtrate was extracted with EtOAc (3 x 50 mL), and the combined organic layers dried over Na_2SO_4 and evaporated to yield the desired product as a slightly pink solid (1.62 g, 73% isolated yield). The product could be purified further by silica gel chromatography eluting with a gradient of 0→60% EtOAc in heptanes, but the crude product was sufficiently pure to carry on to the next step. ^{1}H NMR (300 MHz, DMSO-d_6) δ 9.22 (br s, 1H); 7.80 (br s, 1H); 7.46 (d, J = 8.8 Hz, 1H); 7.40 (d, J = 8.8 Hz, 1 H); 7.30 (dd, J = 8.8, 2.2 Hz, 1H); 6.89 (dd, J = 8.8, 2.2 Hz, 1H); 6.76 (d, J = 2.2 Hz, 1H); 5.18 (br s, 2H); 1.50 (s, 9 H); ^{13}C NMR (75 MHz, $CDCl_3$) δ 153.0; 143.1; 132.9; 131.5; 128.6; 128.3; 126.5; 120.0; 118.9; 115.3; 108.6; 80.4; 28.4; LC-MS (ESI) m/z = 259.2 $[M + H]^+$.

***tert*-Butyl (6-amino-5-bromonaphthalen-2-yl)carbamate (S3).** *tert*-Butyl (6-aminonaphthalen-2-yl)carbamate (**S2**, 1.31 g, 5.07 mmol) was stirred with ammonium acetate (39 mg, 0.50 mmol) in MeCN (20 mL) in an ice bath. To the solution, was added *N*-Bromosuccinimide (948 mg, 5.32 mmol) in MeCN (10 mL) dropwise over 1 h. The reaction was stirred for 1 h at 0 °C, and then loaded onto Celite under reduced pressure. The product was isolated as a foam (1.33 g, 78% isolated yield) by silica gel chromatography eluting with a gradient of 0→50% EtOAc in heptanes. ^{1}H NMR (300 MHz, CD_2Cl_2) δ 7.94 (dd, J = 9.0, 0.6 Hz, 1H); 7.89 (br d, J = 0.6 Hz, 1H); 7.58 (dt, J = 8.8, 0.6 Hz, 1H); 7.38 (dd, J = 9.0, 2.3 Hz, 1H); 7.02 (d, J = 8.8 Hz, 1H); 6.66 (br s, 1 H); 4.35 (br s, 2 H); 1.54 (s, 9 H); ^{13}C NMR (75 MHz, $CDCl_3$) δ 152.9; 141.0; 133.5; 129.6; 129.0; 128.0; 125.8; 120.8; 118.4; 115.4; 104.1; 80.6; 28.4; LC-MS (ESI) m/z = 339.1 $[M + H]^+$.

***tert*-Butyl (2-cyanonaphtho[2,1-d]thiazol-7-yl)carbamate (S4).** To a stirred solution of *tert*-butyl (6-amino-5-bromonaphthalen-2-yl)carbamate (**S3**, 540 mg, 1.6 mmol) in dry DCM (20 mL), was added Appel's salt (351 mg, 1.68 mmol) in one portion. Upon completion, the reaction was diluted with EtOAc (100 mL) and washed with water (20 mL). The organic layer was dried over Na_2SO_4 and evaporated. The crude material thus obtained was dissolved in 10 mL of pyridine and treated with CuI (529 mg, 2.78 mmol). The mixture was heated at 110 °C for 40 min, and concentrated in vacuo. The resulting solids were taken up in EtOAc (100 mL) and filtered. The filtrate was washed with 1 N aqueous HCl solution (20 mL), the aqueous layer extracted with EtOAc (2 x 20 mL), and the combined organic layers dried over Na_2SO_4. The solvents were removed under reduced pressure, and the resulting material subjected to

silica gel chromatography eluting with 15→35% EtOAc in heptanes to yield the desired product as a yellow solid (148 mg, 30% over 2 steps). ^{1}H NMR (300 MHz, $CDCl_3$) *δ* 8.21 (br s, 1H); 8.07 (d, *J* = 9.0 Hz, 1H); 7.96 (d, *J* = 8.8 Hz, 1H); 7.89 (d, *J* = 9.0 Hz, 1H); 7.54 (d, *J* = 8.8 Hz, 1H); 6.80 (br s, 1H); 1.55 (s, 9H); ^{13}C NMR (75 MHz, $CDCl_3$) *δ* 152.5; 150.2; 138.3; 134.4; 133.7; 133.3; 129.0; 126.3; 123.0; 122.6; 120.3; 115.8; 113.3; 81.4; 28.3; LC-MS (ESI) *m/z* = 326.1 $[M + H]^+$.

(*S*)-2-(7-Aminonaphtho[2,1-d]thiazol-2-yl)-4,5-dihydrothiazole-4-carboxylic acid (NH_2-NpLH2). To a stirred solution of *tert*-butyl (2-cyanonaphtho[2,1-d]thiazol-7-yl)carbamate (**S4**, 72 mg, 0.22 mmol) and thioanisole (0.5 mL) in DCM (2 mL) in an ice bath, was added trifluoroacetic acid (2 mL). After 1 h, the reaction was concentrated under reduced pressure, and the product, 7-aminonaphtho[2,1-d]thiazole-2-carbonitrile (47 mg, 94% isolated yield) was isolated by silica gel chromatography eluting with 0→75% EtOAc in heptanes. ^{1}H NMR (300 MHz, CD_2Cl_2) *δ* 8.00 (d, *J* = 9.0 Hz, 1H); 7.88 (dd, *J* = 8.5, 0.6 Hz, 1H); 7.75 (dt, *J* = 9.0, 0.6 Hz, 1H); 7.09 – 7.16 (m, 2H); LC-MS (ESI) *m/z* = 226.1 $[M + H]^+$.

A solution of 7-aminonaphtho[2,1-d]thiazole-2-carbonitrile (24 mg, 0.106 mmol) in acetonitrile (4 mL) was treated with an aqueous solution (2 mL) containing *D*-cysteine (41 mg, 0.233 mmol) and K_2CO_3 (45 mg, 0.326 mmol). After stirring at ambient temperature for 20 min, the reaction was directly loaded onto preparative HPLC and the product was isolated by eluting with a gradient of 5→100% MeCN in 10 mM aqueous NH_4OAc. The appropriate fractions were concentrated under reduced pressure and then lyophilized to afford the product in free acid form as a yellow solid. The luciferins in free acid form were reconstituted in an aqueous solution basified by sat. aqueous K_2CO_3 solution to pH ~8 –9 and the K salt were isolated by preparative HPLC eluting with 10 min 100% H_2O and 0→100% MeCN in H_2O over 10 min (11 mg, brown powder, 27% isolated yield over 2 steps). m.p. 190 °C (dec.); ^{1}H NMR (300 MHz, D_2O) *δ* 7.47 – 7.20 (m, 3H); 7.04 – 6.84 (m, 2H); 5.14 (dd, *J* = 9.8, 8.1 Hz, 1H); 3.89 – 3.67 (m, 1H); 3.65 – 3.48 (m, 1H). ^{13}C NMR (75 MHz, D_2O/CD_3CN) *δ* 177.1; 164.4; 159.5; 156.2; 147.0; 145.3; 132.9; 132.2; 126.4; 125.6; 119.8; 119.7; 110.9; 79.7; 36.7; HRMS (ESI+) calcd for $C_{15}H_{12}N_3O_2S_2^+$ $[M + Na]^+$: m/z 352.0185, found 352.0188 (Δ 0.9 ppm).

1-Bromo-6-methoxy-2-naphthylamine (S6). 6-Methoxy-2-naphthylamine (**S5**, 400 mg, 1.6 mmol) was suspended in MeCN (20 mL) with NH_4OAc (12 mg, 0.16 mmol). To the mixture stirred in an ice bath, was added *N*-Bromosuccinimide (299 mg, 1.68 mmol) in MeCN (6 mL) dropwise slowly over 1 h. The reaction was stirred for 1 h at 0 °C, adsorbed onto Celite, concentrated in vacuo and subjected to silica gel chromatography eluting with 0→60% EtOAc in heptanes to yield the product as an orange solid (300 mg,

75% isolated yield). ^1H NMR (300 MHz, CD_2Cl_2) δ 7.92 (dt, J = 9.2, 0.7 Hz, 1H); 7.54 (dt, J = 8.7, 0.7 Hz, 1H); 7.22 – 7.10 (m, 1H); 7.07 (d, J = 2.6 Hz, 1H); 7.01 (dd, J = 8.7, 0.7 Hz, 1H); 4.29 (br s, 2 H); 3.89 (s, 3 H); ^{13}C NMR (75 MHz, $CDCl_3$) δ 155.7; 140.3; 129.4; 128.4; 127.3; 126.6; 119.8; 118.3; 106.7; 104.7; 55.4; LC-MS (ESI) m/z = 252.0 $[M + H]^+$.

7-Methoxynaphtho[2,1-*d*]thiazole-2-carbonitrile (S7). To a solution of 1-bromo-6-methoxy-2-naphthylamine (**S6**, 290 mg, 1.15 mmol) in DCM (10 mL), was added Appel's salt (252 mg, 1.21 mmol). After stirring for 24 h at ambient temperature, the reaction was diluted with EtOAc (100 mL) and washed with water (50 mL), dried over Na_2SO_4 and adsorbed onto Celite. Silica gel chromatography eluting with 0→40% EtOAc in heptanes afforded the desired adduct, which was taken up in 5 mL of pyridine and treated with CuI (168 mg, 0.882 mmol). The reaction was heated to reflux for 1.5 h, adsorbed onto Celite and subjected to silica gel chromatography eluting with 0→40% EtOAc in heptanes to afford the desired product (63 mg, 23% isolated yield from **S6**). ^1H NMR (300 MHz, $CDCl_3$) δ 8.08 (d, J = 9.0 Hz, 1H); 7.94 (d, J = 9.6 Hz, 1H); 7.87 (d, J = 9.0 Hz, 1H); 7.36 – 7.28 (m, 2H); 3.99 (s, 3 H); ^{13}C NMR (75 MHz, $CDCl_3$) δ 159.5; 149.7; 134.7; 134.0; 133.4; 128.5; 126.9; 122.5; 121.8; 119.9; 113.5; 108.2; 55.6; LC-MS (ESI) m/z = 241.1 $[M + H]^+$.

7-Hydroxynaphtho[2,1-*d*]thiazole-2-carbonitrile (S8). 7-Methoxynaphtho[2,1-*d*]thiazole-2-carbonitrile (**S7**, 88 mg, 0.366 mmol) was suspended in pyridine hydrochloride salt (3 g, 26.0 mmol) and sealed in a 5-mL microwave vial containing a stir bar. The mixture was maintained stirring at 220 °C for 25 min under MW condition, then taken up in 1 N aqueous HCl solution (50 mL) and extracted with DCM/EtOAc (1/1, 3 X 25 mL) . The combined organic layers were concentrated in vacuo, and the crude was purified by silica gel chromatography eluting with 0→40% MeOH in DCM to yield the desired product as yellow solid (67 mg, 81% isolated yield). ^1H NMR (300 MHz, CD_2Cl_2) δ 8.09 (d, J = 9.0 Hz, 1H); 8.02 (d, J = 8.8 Hz, 1H); 7.87 (d, J = 9.0 Hz, 1H); 7.38 (d, J = 2.6 Hz, 1H); 7.29 (dd, J = 8.8, 2.6 Hz, 1H); 5.45 (br s, 1H); ^{13}C NMR (75 MHz, DMF-*d6*) δ 158.6; 149.4; 135.3; 134.7; 133.1; 128.7; 127.6; 122.0; 120.9; 120.2; 113.9; 111.6; LC-MS (ESI) m/z = 227.0 $[M + H]^+$.

(*S*)-2-(7-Hydroxynaphtho[2,1-d]thiazol-2-yl)-4,5-dihydrothiazole-4-carboxylic acid (OH-NpLH2). A solution of 7-hethoxynaphtho[2,1-d]thiazole-2-carbonitrile (**S8**, 35 mg, 0.16 mmol) in MeCN (4 mL) was treated with *D*-cysteine (54 mg, 0.31 mmol) and K_2CO_3 (54 mg, 0.39 mmol) in H_2O (2 mL). After stirring at ambient temperature for 20 min, the reaction was neutralized by addition of AcOH, and the product was

isolated by preparative HPLC eluting with a gradient of 5→95% MeCN in 10 mM aqueous NH_4OAc. The appropriate fractions were concentrated under reduced pressure and then lyophilized. The luciferins in free acid form were reconstituted in an aqueous solution basified by sat. aqueous K_2CO_3 solution to pH ~8–9 and the K salt were isolated by preparative HPLC eluting with 10 min 100% H_2O and 0→100% MeCN in H_2O over 10 min (16 mg, brown powder, 28% isolated yield over 2 steps). m.p. 185 °C (dec.); ^{1}H NMR (300 MHz, D_2O) δ 7.38 – 7.14 (m, 3H); 6.99 – 6.89 (m, 2H); 5.20 (dd, J = 9.9, 8.4 Hz, 1H); 3.85 (dd, J = 11.0, 9.9 Hz, 1H); 3.63 (dd, J = 11.0, 8.4 Hz, 1H). ^{13}C NMR (75 MHz, D_2O/CD_3CN) δ 177.1; 164.4; 156.5; 154.1; 147.1; 132.4; 131.9; 126.4; 126.0; 120.1; 119.8; 117.7; 110.8; 79.8; 35.8; HRMS (ESI+) calcd for $C_{15}H_{11}N_2O_3S_2^+$ [M + H]$^+$: m/z 331.0206, found 331.0213 (Δ 2.1 ppm).

All NMR spectra were recorded on a Varian 300 MHz spectrometer at 300 MHz for 1H and 75 MHz for 13C. 1H NMR spectra were referenced to residual CDCl3 (7.27 ppm), CD2Cl2 (5.32 ppm), DMF-D7 (8.03, 2.92, 2.75 ppm), D2O (4.80 ppm) or DMSO-D6 (2.50 ppm); 13C NMR spectra were referenced to CDCl3 (77.23 ppm) or DMF-D7 (163.15, 34.89, 29.76 ppm). Data for 1H NMR are reported as follows: chemical shift (multiplicity, coupling constant in Hertz (Hz), number of hydrogens). Abbreviations are as follows: br = broad, s = singlet, d = doublet, t = triplet, q = quartet, m = multiplet.
