# Supplementary material for: Click beetle luciferase mutant and near infrared naphthyl-luciferins for improved bioluminescence imaging
Source: Nat Commun. 2018 Jan 9;9:132. doi: 10.1038/s41467-017-02542-9 (PMC5760652; doi:10.1038/s41467-017-02542-9)
Supplement: Supplementary file 3 — Description of Additional Supplementary Files [file 41467_2017_2542_MOESM3_ESM.pdf]

Descriptions of Additional Supplementary Files

File Name: Supplementary Movie 1
Description: Three-dimensional reconstruction of bioluminescent signal from CBR2opt expressing cells upon injection of NH2-naphthyl luciferin.

File Name: Supplementary Movie 2
Description: Three-dimensional reconstruction of bioluminescent signal from CBR2opt expressing cells upon injection of D-luciferin.
